# A DNA Barcoding Approach to Characterize Pollen Collected by Honeybees

**DOI:** 10.1371/journal.pone.0109363

**Published:** 2014-10-08

**Authors:** Andrea Galimberti, Fabrizio De Mattia, Ilaria Bruni, Daniela Scaccabarozzi, Anna Sandionigi, Michela Barbuto, Maurizio Casiraghi, Massimo Labra

**Affiliations:** 1 Università degli Studi di Milano-Bicocca, ZooPlantLab, Dipartimento di Biotecnologie e Bioscienze, Milano, Italy; 2 Parco Regionale della Grigna Settentrionale, Barzio, Italy; Consiglio Nazionale delle Ricerche (CNR), Italy

## Abstract

In the present study, we investigated DNA barcoding effectiveness to characterize honeybee pollen pellets, a food supplement largely used for human nutrition due to its therapeutic properties. We collected pollen pellets using modified beehives placed in three zones within an alpine protected area (Grigna Settentrionale Regional Park, Italy). A DNA barcoding reference database, including *rbcL* and *trnH-psbA* sequences from 693 plant species (104 sequenced in this study) was assembled. The database was used to identify pollen collected from the hives. Fifty-two plant species were identified at the molecular level. Results suggested *rbcL* alone could not distinguish among congeneric plants; however, *psbA-trnH* identified most of the pollen samples at the species level. Substantial variability in pollen composition was observed between the highest elevation locality (Alpe Moconodeno), characterized by arid grasslands and a rocky substrate, and the other two sites (Cornisella and Ortanella) at lower altitudes. Pollen from Ortanella and Cornisella showed the presence of typical deciduous forest species; however in samples collected at Ortanella, pollen of the invasive *Lonicera japoni*ca, and the ornamental *Pelargonium x hortorum* were observed. Our results indicated pollen composition was largely influenced by floristic local biodiversity, plant phenology, and the presence of alien flowering species. Therefore, pollen molecular characterization based on DNA barcoding might serve useful to beekeepers in obtaining honeybee products with specific nutritional or therapeutic characteristics desired by food market demands.

## Introduction

The current pollen demand for human nutrition has drastically increased due to its therapeutic value, with potential for medical and nutritional applications [1]–[4]. Pollen pellets collected by honeybees (*Apis mellifera* L.) contain proteins, all the basic amino acids, carbohydrates, lipids, such as Omega-3 and Omega-6 fatty acids, vitamins and minerals [5]–[7]. Based on the presence of these compounds, pollen is eligible as human food, and national pollen standards exist in a number of countries [8]. Consequently, many beekeepers have transitioned their interests to pollen collection, and currently, the global pollen production is approximately 1500 tons per year, with Spain the most important producer [9].

Honeybees collect pollen grains during flower visiting, and agglutinate the grains into pellets using a nectar-saliva mixture. Pellets are subsequently transported into hives to feed honeybee larvae. Pollen pellets are collected by beekeepers at the hive entrance using specific pollen traps [10], processed, and stored to be delivered to market.

The pollen load and composition varies substantially in response to different elements influencing honeybee activity, including geographical position of the hive, and annual changes in local flora and flowering phenology [11]. Pollen has specific characteristics associated with the plant species, and pollen nutritional value shows consistent differences among taxa [2], [4]. Moreover, pollen composition and diversity (in terms of plant origin) directly influences the quality and safety of other honeybee products, such as honey, royal jelly, and propolis. The identification of plants visited by honeybees is of fundamental importance for beekeepers to assess the quality of their products, and guarantee the consumer of product safety. In addition, the geographical origin of pollen strongly affects its commercial value [12]–[14].

The conventional means to identify pollen origins is conducted by microscopic analysis, and comparisons with morphological keys [15], [16]. Although this approach is widely adopted, it is time-consuming, requires extensive botanic knowledge, and involves a laborious counting procedure. Furthermore, identification of species using pollen morphological analysis is often unsuccessful [17]. In some cases, pollen shows peculiar morphological traits that make it recognizable (e.g., *Eucalyptus* and *Castanea*), whereas in many cases, differentiating pollen of congeneric species (e.g., in some genera of Campanulaceae and Lamiaceae) with micromorphology is challenging [17], [18].

Molecular-based techniques have shown great potential in overcoming these limitations, as demonstrated by the recent literature [19]–[22]. For example, analysis of ITS regions was successful in identifying plant species visited by Hawaiian bees [23], and to characterize pollen from environmental sediments [24]–[26]. Our objective was to establish a universal and reliable molecular identification system for pollen using DNA barcoding [27]. Recent studies [28], [29] indicated the combination of two plastid regions as ‘barcode’ markers (i.e. *rbcL* and the intergenic spacer *trhH-psbA*) were most effective in achieving maximum universality and highest discrimination power in plants.

Our investigation was conducted on pollen collected by honeybees in the Grigna Settentrionale Regional Park (Lombardy, Northern Italy). This region is one of the richest in flowering species (more than 1,500 vascular plants [30]) throughout Europe, with many rare and endemic taxa.

From a technical point of view, this study aimed to assess the effectiveness of DNA barcoding to identify species from pollen collected by honeybees. Therefore, we selected and characterized a list of plants pollinated by insects in the Grigna Settentrionale Regional Park, including rare and endemic taxa, at the molecular level. The list was used to assemble a reference database of DNA barcoding sequences for taxonomic identification of pollen samples. Moreover, beginning with extensive knowledge of plant phenology in the study area, we also evaluated the effects of local floral biodiversity on bee pollen load in different periods and sub-localities. The final objective was to provide a reliable traceability system, useful for certifying pollen-based products for human consumption.

## Materials and Methods

### Study area and Ethics statement

**Figure 1:**
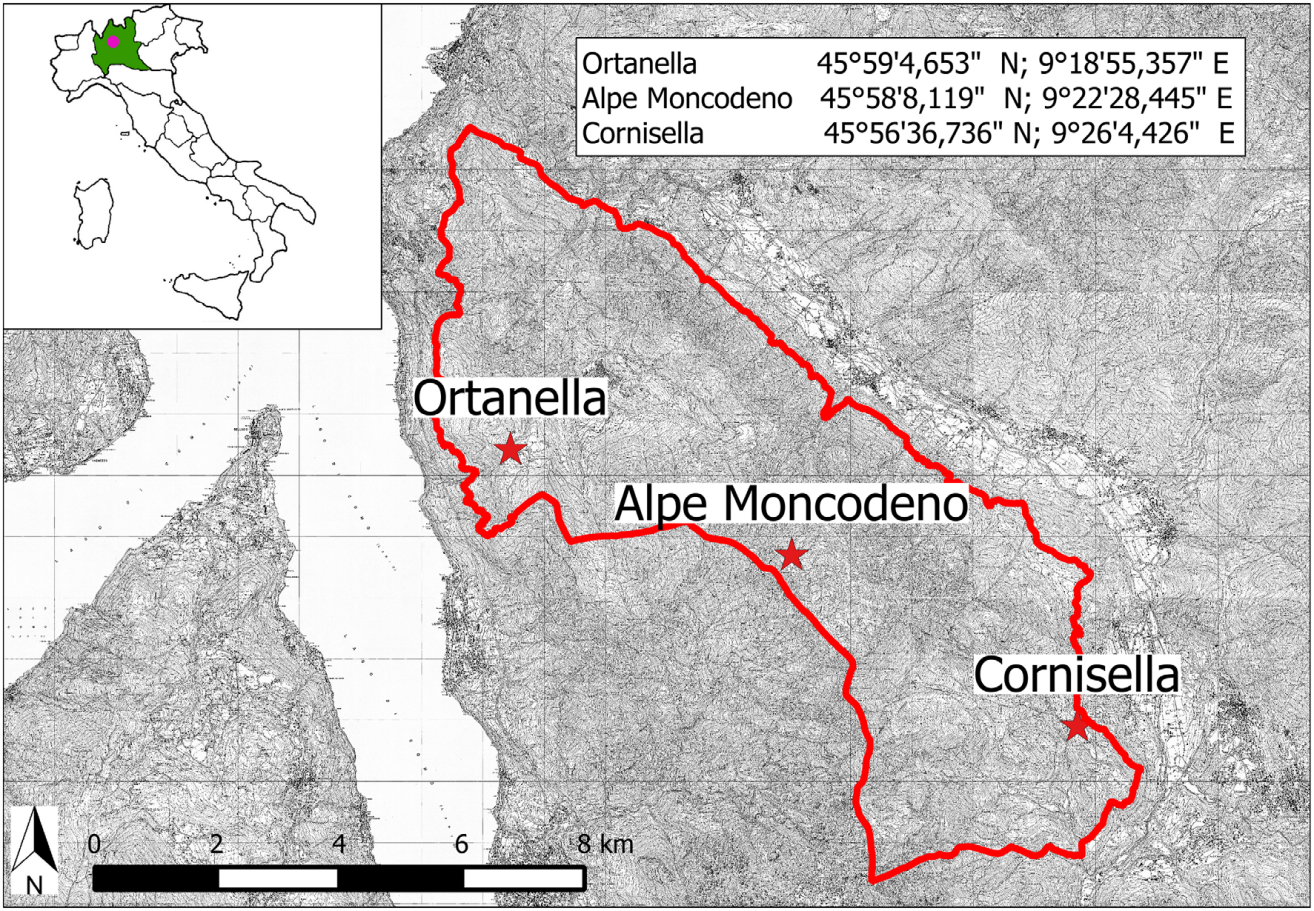
Sampling localities. Distribution map of sites where beehives for pollen collection were positioned within the Grigna Settentrionale Regional Park (red line). The full names and geographic coordinates for the collection sites are provided.

The investigated area is the regional park of Grigna Settentrionale, Northern Italy (Figure 1). It covers a territory of 5,000 hectares around the Grigne massif (Italian Alps). Besides the rather limited altitude (the highest summit reaches 2,409 m), this protected area is characterized by a great variety of habitats and climates ranging from the typical alpine climate to the submediterrean one, because of the nearby Como Lake.

The total flora of this protected area consists of 1,535 vascular plants, including cultivated species and many rare (i.e., patchy-distributed species) or endemic taxa [30]. The vegetation of the park varies according to the altitude: the lowest slopes up to 900 m are covered by forests of hornbeam (*Carpinus*), oak (*Quercus*), chestnut (*Castanea*), ash (*Fraxinus*) and linden (*Tilia*) alternated by pasture meadows. At higher altitudes, up to 1900 m, vegetation is characterized by beech (*Fagus*) and larch (*Larix*) forests. Near the summit, there are heaths with *Rhododendron*, mountain pine (*Pinus mugo* Turra), juniper (*Juniperus*) and green alder (*Alnus viridis* (Chaix.) D.C.), marking the limit of the trees. At the highest altitudes, next to the top of the massif, the area is dominated by grasslands typical of calcareous substrates, characterized by annual species (e.g. *Carex*, *Sesleria*) and chasmophytic vegetation.

Three different localities, hereafter named as Cornisella (lat: 45°56′36.736″N, long: 9°26′4.426″E), Ortanella (lat: 45°59′4.653″N, long: 9°18′55.357″E) and Alpe Moncodeno (lat: 45°58′8.119″N, long: 9°22′28.445″E), were selected as study areas to investigate the diversity of pollen collected by honeybees (Figure 1). Ortanella is located at 950 m above sea level and is characterized by meadows surrounded by a mixed deciduous forest. This locality is very close (about 2 km) to the Lake Como and to Esino Lario, one of the largest urban centres in the park (18 squares kilometers, 750 habitants). Cornisella, located at an altitude of 1000 m has vegetation characteristics similar to Ortanella, but it differs in being less urbanized. The third sampling locality, Alpe Moncodeno, is located at an altitude of 1600 m and is characterized by rare larch forest, distributed sporadically on rocks and dry grasslands.

All experiments, procedures and ethical issues conformed to the competent national ethical bodies. Sampling activities were conducted according to Permesso di Campionamento N°DPN/2D/2004/13650 granted by the Ministero dell’Ambiente della Repubblica Italiana with the approval of the Grigna Settentrionale Regional Park authority. The location was not privately owned and field studies did not compromise the health of endangered or protected plant species.

### Pollen collection and DNA extraction

Pollen pellets were collected from beehives using pollen traps [10]. These devices are equipped with grids that fit across the hive entrance, forcing returning foragers to walk through the grid to enter. The grid scrapes some of the pollen from the corbicula of entering honeybees, and the pellets fall into a tray where they can be easily collected (see Figure S1).

Six modified beehives were placed in three study areas (two beehives at each site) from May to June 2011. Pollen sampling was conducted three times (20 May, 9 June, and 29 June) to span the maximum flowering range of most plants distributed in the park, including rare and endemic taxa [30]. A mixture of approximately 5 g of pollen was collected from the two beehives at each sampling site, and was subsequently stored at −20°C. Material for DNA extraction was obtained by mixing 1 g of pollen pellets collected from each beehive at the same locality (and for each sampling date), freezing the mixture in liquid nitrogen, and grinding it into a fine powder. Genomic DNA was extracted from approximately 100 mg of the pollen mixture using the Plant DNeasy Isolation and Purification kit (Qiagen, Milan, Italy). Purified DNA concentration was estimated for each sample fluorometrically, and by comparing ethidium bromide-stained band intensities with a λ DNA standard on an agarose gel.

### Plant reference molecular database

We assembled a DNA barcoding reference database for Grigna Settentrionale Regional Park plants. The resulting database consisted of 693 species selected from the park’s floristic check-list [30]. The reference database included the most common insect pollinated species surrounding the beehives at a range of 1 km. Endemic and rare species were also included. A complete list of the species selected as reference is provided as supporting information (see Table S1 in Supporting Information).

Among the 693 taxa in the reference database, 104 species were newly characterized with DNA barcoding (*rbcL* and *trnH-psbA*) using fresh samples collected in the park during spring 2011. For the remaining 589 species, *rbcL* and *trnH-psbA* sequences were retrieved from GenBank, where sequences from 258 species, were generated by our group during previous DNA barcoding studies on alpine flora [28], [29]. Records from GenBank were chosen and selected after a careful evaluation of accession characteristics to avoid misidentification in the next bioinformatics analyses (i.e., availability of voucher details and sequence overlapping with those generated in this study).

For each one of the 104 taxa analyzed in this study, a fresh sample (i.e., young leaves or buds) was collected in the park and stored at −20°C. All samples were vouchered as ‘MIB:ZPL’ following the protocol specified by the Global Registry of Biodiversity Repositories (http://grbio.org/), and the data standards for BARCODE Records [31]. Specimens and voucher codes are listed in Table S1. DNA extraction was obtained from 100 mg of fresh tissue, and treated with the Plant DNeasy Isolation and Purification kit (Qiagen, Milan, Italy). PCR conditions used to amplify *rbcL* and *trnH-psbA* regions were the same adopted for pollen samples. PCR products were directly sequenced with both amplification primers, and barcode sequences were submitted to EMBL (Accession numbers: HG416958–HG417061, HG800593 (*rbcL*); HG800488–HG800592 (*trnH-psb*A); see also Table S1).

### Amplification, cloning and sequencing of barcode regions

DNA barcoding analysis was conducted using a portion of the plastidial *rbcL* gene and the *trnH-psbA* intergenic spacer. For PCR amplification and sequencing of *rbcL* and *trnH-psbA*, the primer combinations were retrieved from [32] and [33]. PCRs were performed starting from 10 ng of DNA by using puReTaq Ready-To-Go PCR beads (Amersham Bioscience, Freiburg, Germany) in a 25 µL reaction according to manufacturer’s instructions. PCR cycles consisted of an initial denaturation step for 7 min at 94°C, 35 cycles of denaturation (45 s at 94°C), annealing (30 s at 50°C for *rbcL* and 53°C for *trnH-psbA*), extension (1 min at 72°C), and a final extension at 72°C for 7 min.

The amplification products obtained were checked by electrophoresis on 1.5% (w/v) agarose gel and subsequently cloned using the pGEM-T Easy Vector System (Promega Corporation, Madison, WI, USA). Recombinant plasmids were isolated using Miniprep kit (Applied Biosystems, Foster City, CA) and the insert size and DNA concentration were assessed by another gel electrophoresis. For each mixed pollen sample a total of 100 clones were randomly selected and the inserted barcode amplicons were bidirectionally sequenced with M13 primers using an ABI 155 3730XL automated sequencer at Macrogen Inc., Korea. Raw traces were manually edited with Bioedit v. 7.2.5 [34] and forward and reverse runs were aligned and assembled using Clustal W v. 2.1 [35]. Finally, the 3′ and 5′ terminals were clipped to generate consensus sequences for each accession. In order to avoid the inclusion of inadvertently amplified nuclear pseudogenes of plastidial origin [36], barcode sequences were checked following the guidelines proposed by [37] and [38].

### Taxonomic assignment of pollen samples

Pollen composition was defined by aligning the 100 sequences generated for each pollen mixture with Clustal W 2.1 [35], and subsequent analysis with MEGA 5.1 [39]. Sequences were grouped into MOTUs (Molecular Operational Taxonomic Units) basing on sequence identity, and compared with the reference database using a BLASTn algorithm [40]. Each MOTU was taxonomically assigned to the species showing the nearest matches (maximum identity) according to [28]. Identification results were provided as a list of the nearest matches (maximum identity). When the value of identity matches was lower than 99%, the MOTU was considered ‘unidentifiable’ according to BOLD-IDS guidelines [41]. In these cases, we also performed a BLASTn analysis against the NCBI database. The analysis was performed separately for both the examined markers.

To assess whether the amount of clones sequenced per sampling site was high enough to detect the great majority of pollen sequences, accumulation curves with 1,000 iterations were carried out for each sampling site and for both DNA barcoding markers, with EstimateS 8.2.0 [42]. For each marker, and for each sampling site, the cloned sequences obtained for the three collection periods, were pooled together to exclude any interference of differences in plant phenology among sites.

## Results

### Molecular characterization of pollen mixtures

The DNA extracted from pollen mixtures and plant reference samples showed high quality characteristics, and an acceptable concentration (>20 ng/µl for each sample). High DNA amplification success, with standard primer pairs and thermal conditions was obtained for *rbcL* and *trnH-psbA*. The 104 plant species collected in the Grigna Settentrionale regional park constitute the first DNA barcoding entries in GenBank. These were used to assemble the reference database consisting of 693 taxa. The pollen mixtures collected from beehives at the three study localities on the three sampling dates resulted in identification of 12–18 MOTUs composed of one to 28 sequences showing small nucleotide divergence. These differences were all single-nucleotide substitutions (1–2 nucleotides per clone), occurring at the same position in the DNA sequence. Our analyses did not provide any evidence for the presence of pseudogene or numt interference in the barcode sequences obtained from the samples. Maximum length of aligned sequences was 627 bp and 634 bp for *rbcL* and *trnH-psbA* respectively.

MOTUs were compared to the reference database to identify plant composition from each pollen mixture. Results are shown in Table 1–3 and Table 4. In several cases (i.e., in the genera *Aquilegia*, *Campanula*, *Hypericum*, *Potentilla*, *Prunella, Rubus*, *Salvia,* and *Trifolium*), BLAST analysis performed with *rbcL* did not identify a species with more than 99% certainty (Table 1–3). In contrast, DNA barcoding analysis performed with *trnH-psbA* identified all plant species in pollen samples with some rare exceptions, namely *Acer campestre* L., *A. platanoides* L., *Achillea millefolium* L., *A. clavenna* L., *Quercus pubescens* Willd., *Q. petraea* (Mattuschka) Liebl., *Q. robur* L., *Rhododendron hirsutum* L., and *R. ferrugineum* L. For both markers some unidentified MOTUs were detected (maximum identity values <99%) both for the reference and NCBI database. However, in almost all cases, the DNA barcoding data identified the plant composition for each pollen mixture.

**Table 1:** Molecular identification of pollen mixtures at Cornisella.

	*rbcL* Molecular identification				*trnH-psbA* Molecular identification			
	MOTUs (n. clones)				MOTUs (n. clones)			
Identified plants	SamplingI	SamplingII	SamplingIII	Species matchin ReferenceDatabase (ID %)	SamplingI	SamplingII	SamplingIII	Species match in ReferenceDatabase (ID %)
*Acer* sp.	22	19	_	*Acer campestre* (100) *Acer pseudoplatanus* (99.7) *Acer platanoides* (99.8)	12	16	_	*Acer campestre* (100) *Acer* *platanoides* (99.6)
*Achillea* sp.	_	_	9	*Achillea millefolium* (100) *Tanacetum parthenium* (99.8) *Tanacetum corymbosum* (99.8)	_	2	4	*Achillea millefolium* (100)*Achillea clavennae* (99.5)
*Anthyllis vulneraria*	4	_	_	*Anthyllis vulneraria* (100)	_	_	_	_
*Campanula scheuchzeri*	_	_	10	*Campanula scheuchzeri* (100) *Campanula rotundifolia* (99.4) *Campanula cochleariifolia* (99.4)	_	_	7	*Campanula scheuchzeri* (100)
*Centaurea nigriscens*	_	3	2	*Centaurea nigriscens* (100) *Phyteuma scheuchzeri* (99.4) *Phyteuma ovatum* (99.4)	_	2	7	*Centaurea nigriscens* (100)
*Cirsium erisithales*	_	_	2	*Cirsium erisithales* (100)	_	_	_	_
*Cyanus triumfettii*	4	_	_	*Cyanus triumfettii* (100) *Saussurea alpina* (100) *Phyteuma betonicifolium* (99.8)	1	1	_	*Cyanus triumfettii* (100)
*Fagus sylvatica*	19	11	4	*Fagus sylvatica* (100)	24	19	7	*Fagus sylvatica* (100)
*Geranium robertianum*	1	6	11	*Geranium robertianum* (100)	4	2	17	*Geranium robertianum* (100)
*Hypericum perforatum*	5	5	_	*Hypericum perforatum* (100) *Hypericum maculatum* (99.8) *Hypericum humifusum* (99.1)	2	1	_	*Hypericum perforatum* (100)
*Lotus corniculatus*	13	_	_	*Lotus corniculatus* (100)	8	_	_	*Lotus corniculatus* (100)
*Phyteuma scheuchzeri*	_	1	3	*Phyteuma scheuchzeri* (100) *Centaurea nigriscens* (99.4)	_	3	9	*Phyteuma scheuchzeri* (100)
*Potentilla erecta*	2	_	_	*Potentilla erecta* (100) *Potentilla reptans* (100)	6	_	_	*Potentilla erecta* (100)
*Prunella grandiflora*	8	9	8	*Prunella grandiflora* (100) *Prunella vulgaris* (99.6)	2	11	3	*Prunella grandiflora* (100)
*Rosa canina*	_	9	11	*Rosa canina* (100) *Lilium bulbiferum croceum* (99.7)	2	7	4	*Rosa canina* (100)
*Rubus ulmifolius*	7	5	7	*Rubus ulmifolius* (100) *Rubus caesius* (100)	17	1	9	*Rubus ulmifolius* (100)
*Salvia pratensis*	_	9	_	*Salvia pratensis* (100) *Salvia officinalis* (99.7) *Mentha pulegium* (99.7)	_	16	_	*Salvia pratensis* (100)
*Teucrium montanum*	_	_	_	_	_	_	2	*Teucrium montanum* (100)
*Tragopogon pratensis*	_	5	13	*Tragopogon pratensis* (100) *Cirsium erisithales* (99.4)	5	3	6	*Tragopogon pratensis* (100)
*Trifolium montanum*	9	16	18	*Trifolium montanum* (100) *Trifolium repens* (99.8)	15	12	22	*Trifolium montanum* (100)
*Xerolekia speciosissima*	_	_	_	*_*	_	3	_	*Xerolekia speciosissima* (100)
Unidentifiable	6	2	2	_	2	1	3	_

Molecular identification of the pollen collected at Cornisella (Grigna Settentionale Regional Park) on three different times (May 20th, 2011: Sampling I; June 9th, 2011: Sampling II and June 29th, 2011: Sampling III). The number of clones for each MOTU, the species match in reference database and the related identity values higher than 99% (ID%) obtained with the BLAST search are reported for the two barcode regions.

**Table 2:** Molecular identification of pollen mixtures at Ortanella.

	*rbcL* Molecular identification				*trnH-psbA* Molecular identification			
	MOTUs (n. clones)				MOTUs (n. clones)			
Identified plants	Sampling I	Sampling II	Sampling III	Species match in Reference Database(ID %)	Sampling I	Sampling II	Sampling III	Species match inReference Database(ID %)
*Acer* sp.	5	4	_	*Acer campestre* (100) *Acer* *pseudoplatanus* (99.7) *Acer* *platanoides* (99.8)	9	6	_	*Acer campestre* (100) *Acer platanoides* (99.6)
*Amelanchier* *ovalis*	4	_	_	*Amelanchier ovalis* (100) *Cotoneaster integerrimus* (100) *Crataegus monogyna*(100)	2	3	_	*Amelanchier ovalis* (100)
*Anthyllis* *vulneraria*	_	_	_	_	1	_	_	*Anthyllis vulneraria* (100)
*Aquilegia atrata*	_	_	_	*_*	_	_	2	*Aquilegia atrata* (100)
*Aquilegia* *brauneana*	_	4	2	*Aquilegia* *brauneana* (100) *Aquilegia* *atrata* (100)	_	1	4	*Aquilegia brauneana* (100)
*Astrantia major*	_	3	_	*Astrantia* *major* (100)	_	_	_	_
*Bupleurum* *petraeum*	_	_	_	_	_	_	1	*Bupleurum petraeum* (100)
*Centaurea jacea*	_	_	_	_	_	1	_	*Centaurea jacea* (100)
*Cytisus nigricans*	2	_	_	*Cytisus* *nigricans* (100)	_	_	_	*_*
*Dactylorhiza* *maculata*	_	6	_	*Dactylorhiza* *maculata* (100)*Gymnadenia* *rhellicani*(100) *Traunsteinera* *globosa* (99.8)	_	2	_	*Dactylorhiza maculata* (100)
*Fagus sylvatica*	19	17	19	*Fagus* *sylvatica* (100)	14	23	21	*Fagus sylvatica* (100)
*Fraxinus* *excelsior*	5	_	_	*Fraxinus* *excelsior* (100) *Fraxinus ornus* (100) *Ligustrum* *lucidum* (99.6)	3	_	_	*Fraxinus excelsior* (100)
*Genista radiata*	_	_	5	*Genista* *radiata* (100) *Laburnum* *anagyroides* (99.4)	_	_	1	*Genista radiata* (100)
*Geranium robertianum*	2	4	6	*Geranium* *robertianum* (100)	5	_	3	*Geranium robertianum* (100)
*Hippocrepis* *comosa*	3	1	_	*Hippocrepis* *comosa* (100)	_	2	_	*Hippocrepis comosa* (100)
*Horminum* *pyrenaicum*	_	_	_	_	_	_	1	*Horminum pyrenaicum* (100)
*Leontodon* *hispidus*	5	_	6	*Leontodon* *hispidus* (100)*Picris* *hieracioides* *spinulosa* (99.5)	8	3	14	*Leontodon hispidus* (100)
*Lonicera* *japonica*	_	_	5	*Lonicera japonica* (100)	_	_	1	*Lonicera japonica* (100)
*Lotus* *corniculatus*	2	9	3	*Lotus* *corniculatus* (100)	8	7	_	*Lotus corniculatus* (100)
*Pelargonium×hortorum*	4	8	14	*Pelargonium ×* *hortorum* (100)	7	11	19	*Pelargonium×hortorum* (100)
*Physoplexis comosa*	_	_	_	_	_	_	4	*Physoplexis comosa* (100)
*Phyteuma scheuchzeri*	_	5	2	*Phyteuma* *scheuchzeri* (100) *Centaurea* *nigriscens* (99.4)	_	10	5	*Phyteuma scheuchzeri* (100)
*Potentilla erecta*	_	_	3	*Potentilla* *erecta* (100) *Potentilla* *reptans* (100)	3	_	1	*Potentilla erecta* (100)
*Prunella* *grandiflora*	_	1	3	*Prunella grandiflora* (100) *Prunella vulgaris* (99.6)	_	6	7	*Prunella grandiflora* (100)
*Quercus* sp.	24	28	_	*Quercus* *pubescens* (100) *Quercus* *robur* (100)*Quercus suber* (100)	17	19	_	*Quercus pubescens* (100) *Quercus robur* (99.8) *Quercus petraea* (99.1)
*Rosa canina*	4	_	_	*Rosa canina* (100) *Lilium* *bulbiferum* *croceum* (99.7)	_	1	_	*Rosa canina* (100)
*Rubus ulmifolius*	4	_	4	*Rubus* *ulmifolius* (100) *Rubus* *caesius* (100)	8	_	1	*Rubus ulmifolius* (100)
*Salvia pratensis*	_	_	_	*_*	_	2	_	*Salvia pratensis* (100)
*Sanguisorba* *minor*	_	_	1	*Sanguisorba* *minor* (100)	_	_	_	*_*
*Sorbus aria*	_	_	_	*_*	4			*Sorbus aria* (100)
*Trifolium montanum*	15	7	22	*Trifolium montanum* (100) *Trifolium repens* (99.8)	7	3	12	*Trifolium montanum* (100)
Unidentifiable	2	3	5	_	4	_	3	_

Molecular identification of the pollen collected at Ortanella (Grigna Settentionale Regional Park) on three different times (May 20th, 2011: Sampling I; June 9th, 2011: Sampling II and June 29th, 2011: Sampling III). The number of clones for each MOTU, the species match in reference database and the related identity values higher than 99% (ID%) obtained with the BLAST search are reported for the two barcode regions.

**Table 3:** Molecular identification of pollen mixtures at Alpe Moncodeno.

	*rbcL* Molecular identification				*trnH-psbA* Molecular identification			
	MOTUs (n. clones)				MOTUs (n. clones)			
Identified plants	Sampling I	Sampling II	Sampling III	Species match in Reference Database (ID %)	Sampling I	Sampling II	Sampling III	Species match in Reference Database (ID %)
*Aquilegia brauneana*	_	7	_	*Aquilegia brauneana* (100) *Aquilegia atrata* (100)	_	11	2	*Aquilegia brauneana* (100)
*Buphthalmum salicifolium*	_	_	2	*Buphthalmum salicifolium* (100) *Inula britannica* (99.8)	_	_	1	*Buphthalmum salicifolium* (100)
*Campanula cochleariifolia*	_	_	12	*Campanula cochleariifolia* (100) *Campanula rotundifolia* (99.6) *Campanula scheuchzeri* (99.4)	_	_	3	*Campanula cochleariifolia* (100)
*Campanula rainieri*	_	1	2	*Campanula raineri* (100) *Campanula elatinoides* (100) *Campanula cochleariifolia* (99.4)	_	4	9	*Campanula raineri* (100)
*Campanula scheuchzeri*	_	_	5	*Campanula scheuchzeri* (100) *Campanula rotundifolia* (99.4) *Campanula cochleariifolia* (99.4)	_	_	1	*Campanula scheuchzeri* (100)
*Centaurea rhaetica*	_	_	7	*Centaurea rhaetica* (100) *Centaurea jacea* (100) *Phyteuma betonicifolium* (100)	_	_	11	*Centaurea rhaetica* (100)
*Clinopodium alpinum*	_	_	_	_	2		_	*Clinopodium alpinum* (100)
*Cyanus triumfettii*	8	_	_	*Cyanus triumfettii* (100) *Saussurea alpina* (100) *Phyteuma betonicifolium* (99.8)	11	1	_	*Cyanus triumfettii* (100)
*Dactylorhiza maculata*	_	3	2	*Dactylorhiza maculata* (100) *Gymnadenia rhellicani* (100) *Traunsteinera globosa* (99.8)	3	5	2	*Dactylorhiza maculata* (100)
*Fagus sylvatica*	19	_	_	*Fagus sylvatica* (100)	24	_	_	*Fagus sylvatica* (100)
*Genista radiata*	_	3	6	*Genista radiata* (100) *Laburnum anagyroides* (99.4)	_	_	1	*Genista radiata* (100)
*Horminum pyrenaicum*	_	_	_	_	_	_	4	*Horminum pyrenaicum* (100)
*Minuataria grignensis*	_	_	7	*Minuataria grignensis* (100)	_	_	5	*Minuataria grignensis* (100)
*Noccaea rotundifolia grignensis*	_	_	_	_	_	_	1	*Noccaea rotundifolia grignensis* (100)
*Parnassia palustris*	6	3	_	*Parnassia palustris* (100)	1	5	_	*Parnassia palustris* (100)
*Primula grignensis*	9	_	_	*Primula grignensis* (100) *Primula glaucescens* (100)	7	_	_	*Primula grignensis* (100)
*Prunella grandiflora*	6	_	4	*Prunella grandiflora* (100) *Prunella vulgaris* (99.6)	_	1	9	*Prunella grandiflora* (100)
*Rhododendron* sp.	9	18	19	*Rhododendron hirsutum* (100) *Rhododendron ferrugineum* (100)	6	22	12	*Rhododendron hirsutum* (100) *Rhododendron ferrugineum* (99.7)
*Saxifraga aizoides*	_	_	_	*_*	_	1	_	*Saxifraga aizoides (100)*
*Saxifraga caesia*	_	11	4	*Saxifraga caesia* (100) *Saxifraga rotundifolia* (99.6) *Saxifraga aizoides* (99.4)	4	7	11	*Saxifraga caesia* (100)
*Teucrium montanum*	19	17	_	*Teucrium montanum* (100) *Allium insubricum* (99.4) *Teucrium chamaedrys* (99.4)	12	26	_	*Teucrium montanum* (100)
*Trifolium montanum*	13	24	9	*Trifolium montanum* (100) *Trifolium repens* (99.8)	18	8	11	*Trifolium montanum* (100)
*Xerolekia speciosissima*	5	9	20	*Xerolekia speciosissima* (100) *Inula salicina* (99.8) *Buphthalmum salicifolium* (99.6)	8	7	15	*Xerolekia speciosissima* (100)
unidentifiable	6	4	1	_	4	2	2	_

Molecular identification of the pollen collected at Alpe Moncodeno (Grigna Settentionale Regional Park) on three different times (May 20th, 2011: Sampling I; June 9th, 2011: Sampling II and June 29th, 2011: Sampling III). The number of clones for each MOTU, the species match in reference database and the related identity values higher than 99% (ID%) obtained with the BLAST search are reported for the two barcode regions.

**Table 4:** Temporal and spatial distribution of plant species identified in pollen pellets collected by honeybees.

				Locality and Sampling date								
				Cornisella			Ortanella			Alpe Moncodeno		
Species	Flowering period	Pollination	Status	I	II	III	I	II	III	I	II	III
*Acer* sp.	IV–V	Anemophilous/Entomophilous	common	+	+		+	+				
*Achillea* sp.	VI–IX	Entomophilous	common		+	+						
*Amelanchier ovalis* Medik	IV–V	Entomophilous	common				+	+				
*Anthyllis vulneraria* L.	IV–VI	Entomophilous	common	+			+					
*Aquilegia atrata* W.D.J. Koch	V–VII	Entomophilous	common						+			
*Aquilegia brauneana* (Hoppe) Jáv.	VI–VII	Entomophilous	rare					+	+		+	+
*Astrantia major* L.	VI–IX	Entomophilous	common					+				
*Buphthalmum salicifolium* L.	VI–VII	Entomophilous	common									+
*Bupleurum petraeum* L.	VII–VIII	Entomophilous	rare						+			
*Campanula cochleariifolia* Lam.	VI–VII	Entomophilous	common									+
*Campanula raineri* Perp.	VI–VIII	Entomophilous	rare								+	+
*Campanula scheuchzeri* Vill.	VII–VIII	Entomophilous	common			+						+
*Centaurea jacea* (Boiss. & Reut.) Gremli	VI–VII	Entomophilous	common					+				
*Centaurea nigrescens* Willd.	VI–VIII	Entomophilous	common		+	+						
*Centaurea rhaetica* Moritzi	VI–VII	Entomophilous	rare									+
*Cirsium erisithales* (Jacq.) Scop.	VI–VIII	Entomophilous	common			+						
*Clinopodium alpinum* (L.)Kuntze	VI–VIII	Entomophilous	common							+		
*Cyanus triumfettii* (All.) Dostál	V–VIII	Entomophilous	common	+	+					+	+	
*Cytisus nigricans* L.	VI–VII	Entomophilous	common				+					
*Dactylorhiza maculata* (L.) Soó	V–VII	Entomophilous	common					+		+	+	+
*Fagus sylvatica* L.	V	Anemophilous	common	+	+	+	+	+	+	+		
*Fraxinus excelsior* L.	III–IV	Anemophilous	common				+					
*Genista radiata* (L.) Scop.	VI–VII	Entomophilous	common						+		+	+
*Geranium robertianum* L.	V–X	Entomophilous	common	+	+	+	+	+	+			
*Hippocrepis comosa* L.	V–VIII	Entomophilous	common				+	+				
*Horminum pyrenaicum* L.	VII–VIII	Entomophilous	common						+			+
*Hypericum perforatum* L.	V–VIII	Entomophilous	common	+	+							
*Leontodon hispidus* L.	VI–X	Entomophilous	common				+	+	+			
*Lonicera japonica* Thunb.	V–IX	Entomophilous	alien						+			
*Lotus corniculatus* L.	IV–IX	Entomophilous	common	+			+	+	+			
*Minuartia grignensis* (Rchb.) Mattf.	VI–VIII	Entomophilous	rare									+
*Noccaea rotundifolia* (L.) Moench *grignensis* F.K. Mey.	VII–VIII	Entomophilous	rare									+
*Parnassia palustris* L.	VI–VIII	Entomophilous	common							+	+	
*Pelargonium × hortorum* L.H. Bailey	I–XII	Entomophilous	alien				+	+	+			
*Physoplexis comosa* (L.) Schur	VII–VIII	Entomophilous	rare						+			
*Phyteuma scheuchzeri* All.	VI–VIII	Entomophilous	common		+	+		+	+			
*Potentilla erecta* (L.) Raeusch.	V–VIII	Entomophilous	common	+			+		+			
*Primula grignensis* Moser	IV–VII	Entomophilous	rare							+		
*Prunella grandiflora* (L.) Scholler	VI–VIII	Entomophilous	common	+	+	+		+	+	+	+	+
*Quercus* sp.	IV–V	Anemophilous	common				+	+				
*Rhododendron* sp.	V–VII	Entomophilous	common							+	+	+
*Rosa canina* L.	V–VII	Entomophilous	common	+	+	+	+	+				
*Rubus ulmifolius* Schott	V–VII	Entomophilous	common	+	+	+	+		+			
*Salvia pratensis* L.	V–VIII	Entomophilous	common		+			+				
*Sanguisorba minor* Scop.	VII–VIII	Entomophilous	common						+			
*Saxifraga aizoides* L.	VI–VIII	Entomophilous	common								+	
*Saxifraga caesia* L.	VI–VIII	Entomophilous	common							+	+	+
*Sorbus aria* (L.) Crantz	V–VI	Entomophilous	common				+					
*Teucrium montanum* L.	V–VIII	Entomophilous	common			+				+	+	
*Tragopogon pratensis* L.	V–VIII	Entomophilous	common	+	+	+						
*Trifolium montanum* L.	V–VIII	Entomophilous	common	+	+	+	+	+	+	+	+	+
*Xerolekia speciosissima* (L.) Anderb.	VI–VII	Entomophilous	rare		+					+	+	+

List of plant species identified trough DNA barcoding in pollen mixtures collected at three distinct sites of the Grigna Settentrionale Regional Park during three sampling periods (May 20th, 2011: I; June 9th, 2011: II and June 29th, 2011: III). For each species, the flowering period (range of months, as stated in [51]), the type of pollination, the status in the study area (as described in [30]) and the presence/absence (+/empty field) for each sampling and for each locality are reported. Plants status (common, rare and alien) has been assigned basing on their distribution within the Grigna Settentrionale Regional Park.

### Comparison of pollen composition among sampling localities and collection dates

The list of plant species identified in pollen mixtures collected from the three localities on the three sampling dates is provided in Table 4. A total of 21 taxa were identified at Cornisella, 31 at Ortanella and 23 at Alpe Moncodeno. Most taxa are entomophilous; however, some anemophilous species such as Fagus sylvatica L., Fraxinus excelsior L. and Quercus spp. were also detected. The accumulation curves analyzing almost 300 sequences per marker and per study area show that our results covered a great part of the specific diversity in pollen collected at modified beehives (Figure S2). The number of clones chosen in this study is balanced in terms of cost-efficiency, as many more clones would have been required to produce a small increase in the number of species detected. Results also suggested that Cornisella and Ortanella shared 12 species. Alpe Moncodeno exhibited a clear difference in plant composition because it shared only six and seven species with Ortanella and Cornisella, respectively.

Results revealed honeybee pollen collection activity is subjected to rapid and continuous changes related to different local plant phenology. In Cornisella, seven species were detected in all three samplings, while an additional seven plants were detected only once. Similarly, at Ortanella, pollen of only six species was always identified, while traces of 13 flowering plants were found during one sampling only. Finally in Alpe Moncodeno, of 23 total species visited by honeybees in the entire period, half (11) were recorded only once, while six were always observed. The pollen composition showed distinct differences at this locality from the first to the third sampling dates (approximately 75% difference), where the number of species detected in the pollen mixture changed from 12 to 16.

Among the 52 plant taxa identified in the entire sampling, nine corresponded to rare and (in some cases) endemic species. At Ortanella, the pollen collected by honeybees contained exotic species, including *Lonicera japonica* Thunb. and *Pelargonium x hortorum* L.H. Bailey.

## Discussion

The results of this study showed the success of DNA barcoding in characterizing the species composition of pollen mixtures collected by honeybees. Overall, the DNA barcoding approach facilitated taxonomic assignment at the species level for almost all pollen mixtures collected in the Grigna Settentrionale Regional Park. Although *rbcL* exhibited limited discrimination power, especially among congeners, *trnH-psbA* analysis resolved taxonomic ‘indecisions’ in most cases. This situation is not surprising given current knowledge regarding identification performance of *rbcL* at the species level [28], [43]. However, the choice of this marker is fundamental to maintain a certain degree of standardization and compatibility with international genetic repositories, as required by CBOL (Consortium for the Barcode of Life) guidelines. Our data suggested DNA barcoding investigations based on *trnH-psbA* were sufficient to characterize pollen biodiversity among study sites. This is a non-coding region, rich in mononucleotide repeats that might cause sequencing errors, and lead to species misidentification. For these reasons, we agree with the DNA Barcoding Plant Working Group protocols, which also recommended the combination of the coding *rbcL* region (and/or *matK*, see critical considerations in [44], [45]) with the *trnH*-*psbA* spacer to achieve reliable plant identification, including closely related taxa.

Compared to micro-morphological investigations, DNA barcoding is a faster and more standardized method to differentiate pollens. This technique is also more suitable for analysis of complex environmental matrices [46]–[48] containing DNA of different species, such as pollen mixtures. DNA barcoding could be combined with HTS (High-Throughput Sequencing) techniques to obtain a larger DNA fragment number. This would allow researchers to process a huge number of pollen samples or pollen-based products with greater analytical depth, and when some kinds of pollen are present in very small quantities [49]. The application of molecular approaches to pollen identification demands the availability of local dedicated databases [50], not only because the BOLD plant database (http://www.boldsystems.org/) is currently poorly populated for plants, but also because local floras are usually characterized by rare and endemic taxa, lacking molecular characterization [29]. Although some MOTUs from our samples remained unclassified, our reference database, composed of new DNA barcoding data for 104 species, contributed new data to GenBank for several plant species.

The species composition from pollen mixtures clearly showed the dominant plants distributed throughout the sampling sites could have a broad influence on honeybee fieldwork. Deciduous forest of beech and maple is the dominant community at Cornisella and Ortanella, and this was reflected in the molecular analysis of pollen mixtures, which showed DNA barcodes of *F. sylvatica* and *Acer* spp. Basing on [51], these species should exhibit the maximum flowering in April and May. However, at Cornisella and Ortanella, several individuals were found in flower also in May and June, respectively (Scaccabarozzi, pers. com.). These observations explained the occurrence of beech and maple pollen for most of the pollen sampling periods. Similarly, the pollen collected at Ortanella showed DNA of *Quercus*, and this was consistent with floristic reports of *Q. pubescens* forests in this area [30].

At Cornisella and Ortanella, honeybees also collected pollen from the same grassland species, namely *Geranium robertianum* L., *Lotus corniculatus* L., and *Tragopogon pratensis* L. These data support the environmental community structure of the two localities, which is characterized by meadows surrounded by deciduous forests. In contrast, the pollen mixtures collected at Alpe Moncodeno showed typical alpine mountain species of arid grasslands and rocky outcrop and substrate areas (e.g., *Rhododendron* spp., *Saxifraga caesia* L., *S. aizoides* L.).

The occurrence of pollen from *Acer, Fagus, Fraxinus* and *Quercus* supports the hypothesis for which pollinators visit occasionally flowers with characteristics of anemophilous species [52]. Most plants are not strictly anemophilous [53] and several studies reported the collection of pollen from *Acer* [54], *Fagus* [55], *Fraxinus* [52], and *Quercus* [56] by different pollinator insects, including honeybees.

Plant phenology also influences pollen diversity collected by honeybees. Although our monitoring was conducted over a short period, we observed notable changes in the flowering of local flora, and consequently in the species composition of pollen collected from beehives. [57] reported pollen from closely related species differed only slightly in its nutritional proprieties, but larger differences (in terms of biochemical composition) were observed among families and orders. Our results demonstrated marked variability in plants visited based on genus and family (i.e., 46 genera and 19 families). This diversity level in pollen sources might result in different protein, carbohydrate, lipid, vitamin, and mineral composition [3], [5]–[7], with expected variation in nutraceutical properties for humans. Therefore, a preliminary evaluation of local plant phenology is essential to plan the collection of specific “curative” pollen. A subsequent molecular characterization can be used to certify pollen composition.

Our results support the utility of DNA barcoding to act as a reliable traceability tool in confirming geographical provenance of pollen-based products. We identified pollen of several endemic or typical plants of the Grigna Settentrionale Regional Park, including *Aquilegia brauneana* (Hoppe) Jáv., *Minuartia grignensis* (Rchb.) Mattf., and *Xerolekia speciosissima* (L.) Anderb. The occurrence of these plants can be considered a clear signature of geographical provenance: a first step to define a Controlled Designation of Origin of food products.

Finally, local biodiversity and environmental alterations could also affect pollen collection. For example, data from Ortanella showed that invasive and ornamental species such as *L. japoni*c*a* and *P. x hortorum* respectively, influenced honeybee pollen collection activities. Thus, exotic species might play a key role in altering pollen composition transported to the beehives. This phenomenon should be taken into consideration in quality assessment of commercialized honeybee products.

In conclusion, our work demonstrates that beekeepers can address their activity to obtain desired honeybee products by combining extensive knowledge on local flora with DNA barcoding analysis. Molecular analysis could also play an important role in food traceability in light of the recent national and international normative on honeybee products (e.g. Brasil-Instrucao Normativa n.3, de 19 de Janeiro de 2001; Bulgary- Bulgarian standard 2567111–91, Poland-PN-R-78893 “Obnóza pylkowe”-Polish legislation for bee-pollen, Switzerland-Swiss Food Manual: Pollen Bienenprodukte, BAG-Swiss Federal Office for Public Health). A recent study clearly shows that DNA barcoding works well also on the characterization of multiflower honey [58]. Such approach can be therefore considered a valid alternative to the longer and more complex palynological and melissopalynological procedures, with several implications for a wide spectrum of other research fields: from ecological studies on plant-pollinator interactions to detailed nutritional investigations on honeybee products.

## Supporting Information

Figure S1 **Pollen collection system.** To collect pollen pellets, two modified beehives (a) for each sampling locality have been equipped with grids (c) for scraping some of the pollen from the corbicula of entering honeybees. Pellets fall into a tray where they can be easily collected at the designed sampling dates (b).
(TIF)

Figure S2 **Accumulation curves.** Diversity of *rbcL* and *trnH-psbA* DNA barcoding sequences detected in pollen pellets from three sampling sites, in relation to number of clones sequenced. The rarefaction curves for sampling sites showed that almost all of these clearly reached the asymptote, which means that the amount of clones sequenced per locality was high enough to detect the great majority of pollen species sequences in it.
(TIF)

Table S1 **Reference database of plant species for the Grigna Settentrionale Regional Park.** For each species included in the list, the provenance of the sample (Genbank or the voucher name of the sample analyzed in this study), the species status in the study area and Genbank accession numbers for both *rbcL* and *trnH-psbA* are provided. * = sequence retrieved from GenBank.
(DOCX)
