# Supplementary figures and images for: A DNA Barcoding Approach to Characterize Pollen Collected by Honeybees
Source: PLoS One. 2014 Oct 8;9(10):e109363. doi: 10.1371/journal.pone.0109363 (PMC4190116; doi:10.1371/journal.pone.0109363)

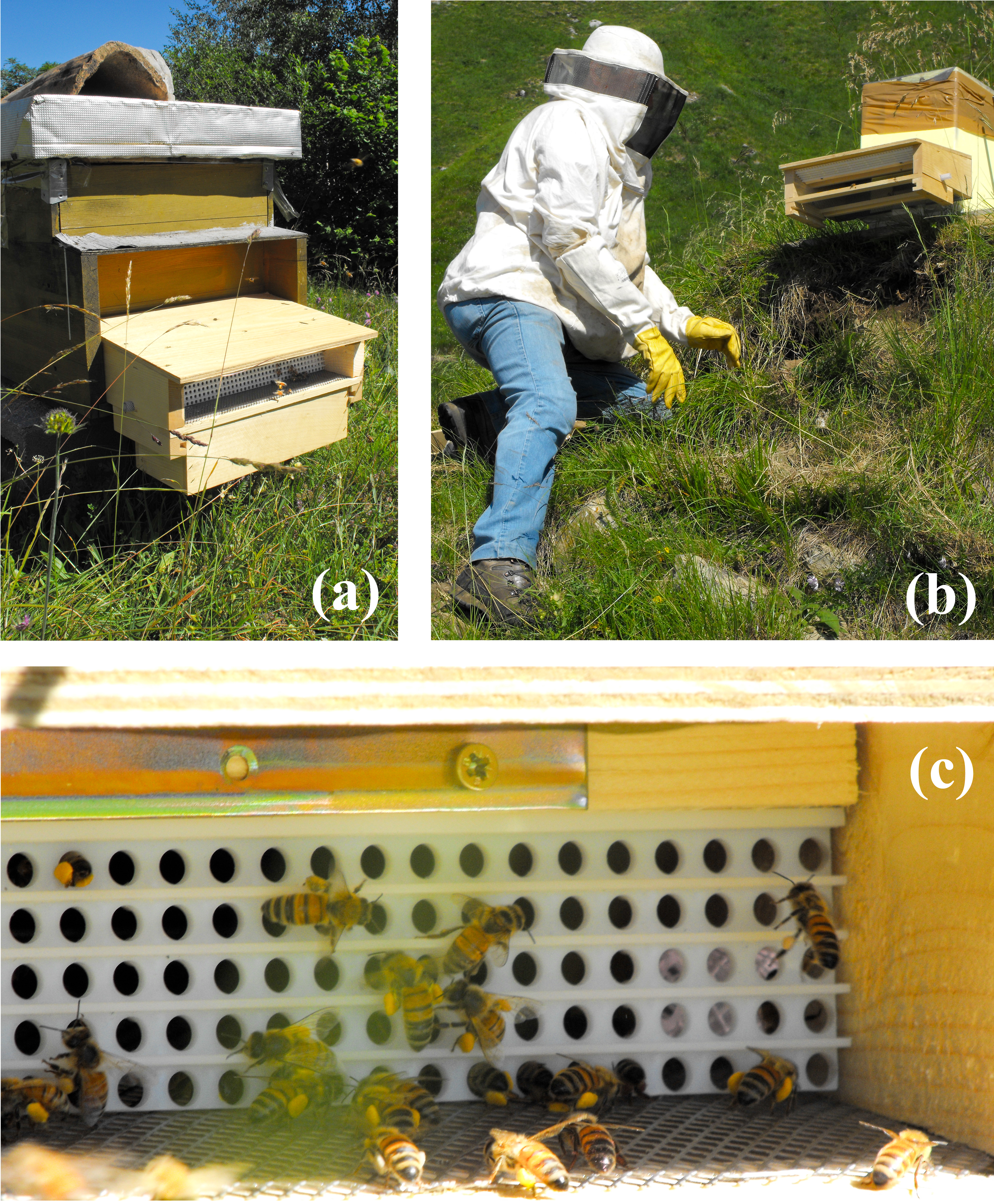

Supplement: Figure S1 — Pollen collection system. To collect pollen pellets, two modified beehives (a) for each sampling locality have been equipped with grids (c) for scraping some of the pollen from the corbicula of entering honeybees. Pellets fall into a tray where they can be easily collected at the designed sampling dates (b). (TIF) [file pone.0109363.s001.tif]

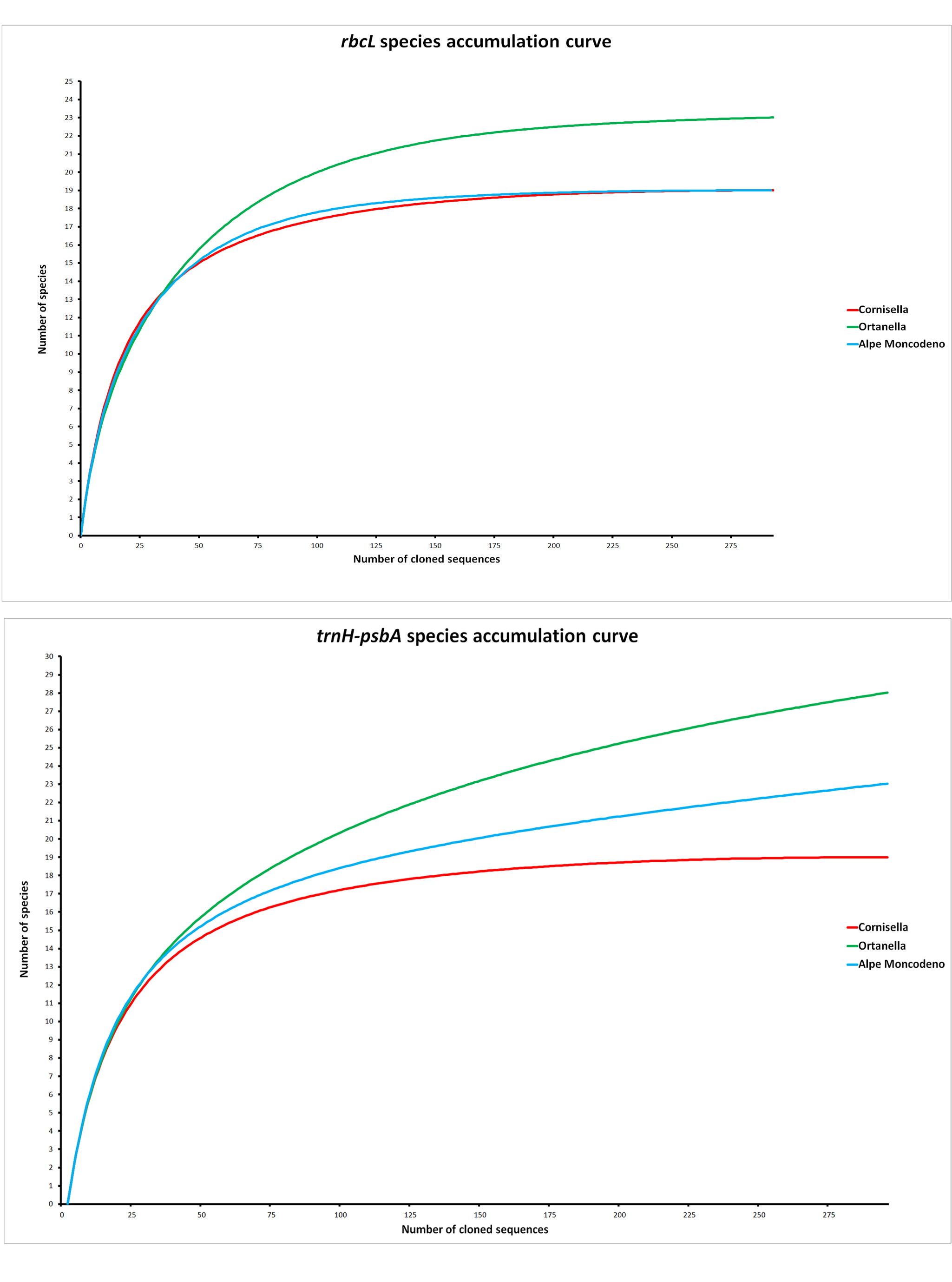

Supplement: Figure S2 — Accumulation curves. Diversity of rbcL and trnH-psbA DNA barcoding sequences detected in pollen pellets from three sampling sites, in relation to number of clones sequenced. The rarefaction curves for sampling sites showed that almost all of these clearly reached the asymptote, which means that the amount of clones sequenced per locality was high enough to detect the great majority of pollen species sequences in it. (TIF) [file pone.0109363.s002.tif]
