# Supplementary material for: A DNA Barcoding Approach to Characterize Pollen Collected by Honeybees
Source: PLoS One. 2014 Oct 8;9(10):e109363. doi: 10.1371/journal.pone.0109363 (PMC4190116; doi:10.1371/journal.pone.0109363)
Supplement: Table S1 — Reference database of plant species for the Grigna Settentrionale Regional Park. For each species included in the list, the provenance of the sample (Genbank or the voucher name of the sample analyzed in this study), the species status in the study area and Genbank accession numbers for both rbcL and trnH-psbA are provided. * = sequence retrieved from GenBank. (DOCX) [file pone.0109363.s003.docx]

**Table S1**

Reference database of plant species for the Grigna Settentrionale Regional Park. For each species included in the list, the provenance of the sample (Genbank or the voucher name of the sample analyzed in this study), the species status in the study area and Genbank accession numbers for both *rbcL* and *trnH-psbA* are provided. * = sequence retrieved from GenBank.

| **Species name** | **Provenance** | **Species status** | ***rbcL*** | ***psbA-trnH*** |
| --- | --- | --- | --- | --- |
| *Abies alba* Mill. | Genbank | Rare | FR831929* | FR832520* |
| *Acanthus mollis* L. | MIB:ZPL:03544 | Alien | HE963302 | HE966457 |
| *Acer campestre* L. | MIB:ZPL:03042 | Common | HE963303 | HE966458 |
| *Acer negundo* L. | MIB:ZPL:03397 | Alien | HE963305 | HE966460 |
| *Acer platanoides* L. | GenBank | Common | FN689356* | FN687504* |
| *Acer pseudoplatanus* L. | MIB:ZPL:03188 | Common | HE963306 | HE966461 |
| *Achillea clavennae* L. | MIB:ZPL:07306 | Common | HG416959 | HG800488 |
| *Achillea millefolium* L. | MIB:ZPL:03502 | Common | HG416958 | HG800489 |
| *Achnatherum calamagrostis* (L.) P. Beauv. | GenBank | Common | JQ933203* | EU204652* |
| *Aconitum napellus* L. emend. Skalický | GenBank | Common | JN892668* | AF216567* |
| *Actaea spicata* L. | Genbank | Common | JQ033710* | JQ033640* |
| *Adiantum capillus-veneris* L. | GenBank | Common | AB574796* | AB575450* |
| *Adoxa moschatellina* L. | Genbank | Common | FJ395577* | FJ395478* |
| *Aegopodium podagraria* L. | Genbank | Common | U50220* | EU445714* |
| *Aesculus hippocastanum* L. | MIB:ZPL:03192 | Alien | HE963308 | HE966463 |
| *Agrostis capillaris* L. | Genbank | Common | JN893043* | FJ395518* |
| *Aira caryophyllea* L. | Genbank | Common | JN893051* | HQ600153* |
| *Ajuga chamaepitys* (L.) Schreb. | Genbank | Common | HQ902771* | HQ902851* |
| *Albizzia julibrissin* Durazz. | Genbank | Common | HQ619748* | EU811952* |
| *Alcea rosea* L. | Genbank | Alien | JQ933211* | EF679744* |
| *Alisma plantago-aquatica* L. | Genbank | Common | JF940702* | JN043786* |
| *Alliaria petiolata* (M. Bieb.) Cavara & Grande | MIB:ZPL:03258 | Common | HE963311 | HE966465 |
| *Allium carinatum* L. | MIB:ZPL:03206 | Common | HE963312 | HE966466 |
| *Allium cepa* L. | MIB:ZPL:06928 | Alien | HF572829 | HF572806 |
| *Allium insubricum* Boiss. & Reut. | MIB:ZPL:07307 | Rare | HG416961 | HG800491 |
| *Allium sativum* L. | MIB:ZPL:06929 | Alien | HF572830 | HF572807 |
| *Allium ursinum* L. | MIB:ZPL:06922 | Common | HF572822 | HF572800 |
| *Allium victorialis* L. | GenBank | Common | HQ690433* | HQ690620* |
| *Allium vineale* L. | MIB:ZPL:03552 | Common | HE963313 | HE966467 |
| *Alnus glutinosa* (L.) Gaertn. | Genbank | Common | FN689372* | FN687522* |
| *Alnus incana* (L.) Moench | Genbank | Common | HM849757* | FJ844534* |
| *Alnus viridis* (Chaix) DC. | GenBank | Common | FJ844588* | FJ844487* |
| *Amaranthus albus* L. | Genbank | Alien | JF940785* | JN043882* |
| *Amaranthus deflexus* L. | MIB:ZPL:03244 | Alien | HE963315 | HE966468 |
| *Amaranthus hybridus* L. | Genbank | Alien | JF940788* | JN043885* |
| *Amaranthus retroflexus* L. | MIB:ZPL:03998 | Alien | HE963314 | HE966469 |
| *Amelanchier ovalis* Medik | MIB:ZPL:03494 | Common | HG416962 | HG800492 |
| *Anagallis arvensis* L. | MIB:ZPL:03191 | Common | HE963319 | HE966473 |
| *Anthericum liliago* L. | MIB:ZPL:03446 | Common | HG416963 | HG800493 |
| *Anthericum ramosum* L. | Genbank/MIB:ZPL:03666 | Common | JX903168* | HE966474 |
| *Anthoxanthum odoratum* L. | MIB:ZPL:03068 | Common | HE963320 | HE966475 |
| *Anthriscus sylvestris* (L.) Hoffm. | Genbank | Common | FJ395576* | FJ395477* |
| *Anthyllis vulneraria* L. | MIB:ZPL:03504 | Common | HG416964 | HG800494 |
| *Antirrhinum majus* L. | MIB:ZPL:03988 | Alien | HE963321 | HE966476 |
| *Aquilegia atrata* W.D.J. Koch | MIB:ZPL:03438 | Common | HG416965 | HG800495 |
| *Aquilegia brauneana* (Hoppe) Jáv. | MIB:ZPL:04286 | Rare | HG416966 | HG800496 |
| *Arabidopsis thaliana* (L.) Heynh. | Genbank | Common | JN891749* | X79898* |
| *Arabis bellidifolia* Crantz | MIB:ZPL:07308 | Rare | HG416967 | HG800497 |
| *Arabis glabra* (L.) Bernh | Genbank | Common | DQ310542* | HQ596600* |
| *Arabis hirsuta* (L.) Scop. | MIB:ZPL:02018 | Common | FR865132 | FR865086 |
| *Arabis sagittata* (Bertol.) DC. | MIB:ZPL:03083 | Common | HE963322 | HE966477 |
| *Arctium lappa* L. | GenBank | Common | JF949994* | GU724258* |
| *Arenaria ciliata* L. | MIB:ZPL:04259 | Common | HG416968 | HG800498 |
| *Aristolochia clematitis* L. | MIB:ZPL:03254 | Common | HE963331 | HE966486 |
| *Arrhenatherum elatius* (L.) P. Beauv. ex J. & C. Presl | MIB:ZPL:04516 | Common | HE963335 | HE966490 |
| *Artemisia absinthium* L. | MIB:ZPL:03238 | Common | HE963336 | HE966491 |
| *Artemisia campestris* L. | GenBank | Common | JN890800* | JN862018* |
| *Artemisia vulgaris* L. | MIB:ZPL:03252 | Common | HE963337 | HE966492 |
| *Arum maculatum* L. | GenBank | Common | JN893086 | FJ395470 |
| *Arundo donax* L. | MIB:ZPL:04514 | Alien | HE963338 | HE966493 |
| Asparagus officinalis L. | Genbank | Common | JN893395* | HM990147* |
| *Asperula aristata* L. f. | MIB:ZPL:03506 | Common | HF567848 | HF567846 |
| *Asperula cynanchica* L. | Genbank/MIB:ZPL:03679 | Common | JN893412* | HE966495 |
| *Asperula purpurea* (L.) Ehrend. | MIB:ZPL:03672 | Common | HE963339 | HE966496 |
| *Asplenium adiantum-nigrum* L. | MIB:ZPL:03051 | Common | HE963340 | HE966497 |
| *Asplenium ruta-muraria* L. | MIB:ZPL:03557 | Common | HE963341 | HE966498 |
| *Asplenium scolopendrium* (Fernald) Kartesz & Gandhi. | GenBank | Common | AB574874* | AB575521* |
| *Asplenium trichomanes* L. | MIB:ZPL:03096 | Common | HE963342 | HE966499 |
| *Asplenium viride* Hudson | Genbank | Common | AB574882* | AB575528* |
| *Aster amellus* L. | MIB:ZPL:02029 | Common | FR865139 | FR865093 |
| *Astragalus alpinus* L. | GenBank | Common | JN965277* | GU338386* |
| *Astrantia major* L. | MIB:ZPL:03483 | Common | HG416969 | HG800499 |
| *Athamanta cretensis* L. | MIB:ZPL:04284 | Common | HG416970 | HG800500 |
| *Athyrium distentifolium* Tausch ex Opiz | Genbank | Common | AB574900* | AB575544* |
| *Athyrium filix-femina* (L.) Roth | GenBank | Common | HQ589969* | HQ157287* |
| *Atocion rupestre* (L.) Oxelman | MIB:ZPL:04310 | Common | HG417049 | HG800579 |
| *Atropa belladonna* L. | MIB:ZPL:07063 | Common | HF572810 | HF572792 |
| *Avena fatua* L. | Genbank | Common | HQ589971* | HQ596610* |
| *Ballota nigra* L. | MIB:ZPL:03255 | Common | HE963348 | HE966505 |
| *Barbarea vulgaris* R. Br. | GenBank | Common | JN893072* | HQ596611* |
| *Bassia scoparia* (L.) A.J. Scott | Genbank | Common | GQ436504* | GQ435156* |
| *Bellis perennis* L. | MIB:ZPL:03201 | Common | HE963349 | HE966506 |
| *Betonica alopecuros* L. | MIB:ZPL:04334 | Common | HG417050 | HG800580 |
| *Betula pendula* Roth | GenBank | Common | JN893300* | GQ248254* |
| *Bistorta officinalis* Delarbre | MIB:ZPL:03512 | Common | HG416971 | HG800501 |
| *Bistorta vivipara* (L.) Delarbre | GenBank | Common | JF943527* | JN046450* |
| *Borago officinalis* L. | MIB:ZPL:03527 | Common | HE963354 | HE966511 |
| *Bothriochloa ischaemum* (L.) Keng | MIB:ZPL:03691 | Common | HE963355 | HE966512 |
| *Botrychium lunaria* (L.) Sw. | Genbank | Common | AB574664* | AB575330* |
| *Brachypodium distachyon* (L.) Beauv. | Genbank | Common | HM849819* | GU575276* |
| *Brachypodium sylvaticum* (Huds.) P. Beauv. | GenBank | Common | HM849820* | HQ600147* |
| *Brassica napus* L. | Genbank | Alien | HQ619737* | FJ493268* |
| *Brassica nigra* Koch | Genbank | Common | HM849822* | AB669924* |
| *Brassica oleracea* L. | Genbank | Alien | JN891540* | FJ493267* |
| *Bromus erectus* Huds. | MIB:ZPL:03093 | Common | HE963358 | HE966515 |
| *Bromus hordeaceus* L. | Genbank | Common | GQ248557* | GQ248255* |
| *Bromus ramosus* Huds. | Genbank | Common | JN892844* | FJ395517* |
| *Bromus sterilis* L. | MIB:ZPL:03075 | Common | HE963359 | HE966516 |
| *Bromus tectorum* L. | Genbank | Common | HQ600441* | HQ600134* |
| *Broussonetia papyrifera* (L.) Vent. | MIB:ZPL:04012 | Alien | HE963360 | HE966517 |
| *Bryonia dioica* Jacq. | Genbank | Common | JN891925* | EU096357* |
| *Buddleja davidii* Franch. | MIB:ZPL:03997 | Common | HE963361 | HE966518 |
| *Buglossoides arvensis* (L.) I.M. Johnst. | Genbank | Common | JN892862* | FJ827357* |
| *Buglossoides purpurocaerulea* (L.) Johnston | MIB:ZPL:03989 | Common | HE963362 | HE966519 |
| *Buphthalmum salicifolium* L. | MIB:ZPL:03493 | Common | HG416972 | HG800502 |
| *Bupleurum falcatum* L. | Genbank | Common | U50224* | JN788913* |
| *Bupleurum petraeum* L. | MIB:ZPL:07309 | Rare | HG416973 | HG800503 |
| *Bupleurum stellatum* L. | MIB:ZPL:04328 | Common | HG416974 | HG800504 |
| *Buxus sempervirens* L. | MIB:ZPL:04006 | Common | HE963368 | HE966525 |
| *Calamagrostis epigejos* (L.) Roth | Genbank | Common | JN892647* | HQ600166* |
| *Calluna vulgaris* (L.) Hull | MIB:ZPL:03062 | Common | HE963372 | HE966529 |
| *Caltha palustris* L. | Genbank | Common | HQ589979* | HQ596617* |
| *Calystegia sepium* (L.) R. Br. *sepium* | Genbank | Common | HQ589980* | HQ596618* |
| *Campanula barbata* L. | MIB:ZPL:07310 | Common | HG416975 | HG800505 |
| *Campanula cochleariifolia* Lam. | MIB:ZPL:04325 | Common | HG416976 | HG800506 |
| *Campanula elatinoides* Moretti | MIB:ZPL:04330 | Rare | HG416977 | HG800507 |
| *Campanula glomerata* L. | MIB:ZPL:03416 | Common | HG416978 | HG800508 |
| *Campanula raineri* Perp. | MIB:ZPL:04324 | Rare | HG416979 | HG800509 |
| *Campanula rapunculoides* L. | GenBank | Common | HQ589981* | HQ596619* |
| *Campanula rapunculus* L. | MIB:ZPL:03194 | Common | HE963374 | HE966531 |
| *Campanula rotundifolia* L. | MIB:ZPL:02019 | Common | FR865133 | FR865087 |
| *Campanula scheuchzeri* Vill. s.l. | MIB:ZPL:03507 | Common | HG416980 | HG800510 |
| *Campanula trachelium*L. | MIB:ZPL:03224 | Common | HE963375 | HE966532 |
| *Cannabis sativa* L. | MIB:ZPL:06919 | Common | HF565316 | HF565307 |
| *Capsella bursa-pastoris* (L.) Medik. | MIB:ZPL:03185 | Common | HE963376 | HE966533 |
| *Capsicum annuum* L. | MIB:ZPL:06933 | Common | HF572816 | HF572795 |
| *Cardamine hirsuta* L. | MIB:ZPL:04002 | Common | HE963379 | HE966536 |
| *Cardamine impatiens* L. | GenBank | Common | JN891994* | JN044165* |
| *Carduus crispus* L. | Genbank | Common | JN893593* | AY914835* |
| *Carduus defloratus* L. | MIB:ZPL:04266 | Common | HG416981 | HG800511 |
| Carex elata *All.* | Genbank | Common | JN893129* | GQ223670* |
| *Carex flacca* Schreb. | MIB:ZPL:03195 | Common | HE963382 | HE966539 |
| *Carex humilis* Leyss | MIB:ZPL:01686 | Common | FR865118 | FR865072 |
| *Carex pairae* F.W. Schultz | MIB:ZPL:03112 | Common | HE963385 | HE966542 |
| *Carlina acaulis* L. | MIB:ZPL:02808 | Common | FR865149 | FR865100 |
| *Carlina vulgaris* L. | Genbank | Common | JN892332* | EU571426* |
| *Carpinus betulus* L. | MIB:ZPL:03338 | Common | HF567847 | HF567845 |
| *Carthamus tinctorius* L. | Genbank | Alien | GQ436451* | GQ435089* |
| *Castanea sativa* Mill. | MIB:ZPL:03568 | Common | HE963387 | HE966544 |
| *Catalpa bignonioides* Walter | MIB:ZPL:03983 | Alien | HE963390 | HE966547 |
| *Cedrus deodora* (Roxburg) Loud. | MIB:ZPL:03108 | Common | HE963394 | HE966551 |
| *Celtis australis* L. | MIB:ZPL:03095 | Common | HE963395 | HE966552 |
| *Centaurea jacea* L. *gaudini* (Boiss. & Reut.) Gremli | MIB:ZPL:01705 | Common | FR865122 | FR865076 |
| *Centaurea nigrescens* Willd. | MIB:ZPL:03484 | Common | HG416982 | HG800512 |
| *Centaurea rhaetica* Moritzi | MIB:ZPL:04265 | Rare | HG416983 | HG800513 |
| *Centaurea scabiosa* L. | GenBank | Common | JN892734* | FJ459726* |
| *Centaurium erythraea* Rafn | GenBank | Common | JN890766* | FJ395452* |
| *Centranthus ruber* (L.) DC. | Genbank | Common | HM849879* | AY794225* |
| *Cephalanthera longifolia* (L.) Fritsch | MIB:ZPL:03444 | Common | HG416984 | HG800514 |
| *Cerastium alpinum* L. | GenBank | Common | JN890578* | AY521425* |
| *Cerastium arvense* L. | Genbank | Common | JN892221* | AY521421* |
| *Cerastium fontanum* Baumg. | Genbank | Common | HQ590026* | HQ596636* |
| *Cerastium glomeratum* Thuill. | Genbank | Common | HQ619745* | FJ493276* |
| *Cerastium semidecandrum* L. | Genbank | Common | JN892588* | AY521444* |
| *Ceratophyllum demersum* L. | Genbank | Common | KC584879* | KC584952* |
| *Cercis siliquastrum* L. | MIB:ZPL:03533 | Common | HE963398 | HE966555 |
| *Ceterach officinarum* Lam. et DC. | MIB:ZPL:03225 | Common | HE963400 | HE966557 |
| *Chaenorhinum minus* (L.) Lange | MIB:ZPL:03239 | Common | HE963401 | HE966558 |
| *Chaerophyllum temulum* L. | MIB:ZPL:03183 | Common | HE963404 | HE966561 |
| *Chamaesyce maculata* (L.) Small | MIB:ZPL:03574 | Alien | HE963406 | HE966563 |
| *Chelidonium majus* L. | MIB:ZPL:03387 | Common | HE963407 | HE966564 |
| *Chenopodium album* L. | Genbank | Alien | JF941269* | JN044292* |
| *Chondrilla juncea* L. | MIB:ZPL:03690 | Common | HE963412 | HE966569 |
| *Chrysopogon gryllus* (L.) Trin. | MIB:ZPL:03229 | Common | HE963413 | HE966570 |
| *Cichorium intybus* L. | MIB:ZPL:03382 | Common | HE963414 | HE966571 |
| *Cirsium arvense* (L.) Scop. | GenBank | Common | JN893242* | FJ395528* |
| *Cirsium erisithales* (Jacq.) Scop. | MIB:ZPL:03510 | Common | HG416985 | HG800515 |
| *Cirsium vulgare* (Savi) Ten. | MIB:ZPL:03665 | Common | HE963416 | HE966573 |
| *Clematis recta* L. | MIB:ZPL:03487 | Common | HG416986 | HG800516 |
| *Clematis vitalba* L. | MIB:ZPL:03102 | Common | HE963422 | HE966575 |
| *Clinopodium alpinum* (L.) Kuntze | MIB:ZPL:04273 | Common | HG416960 | HG800490 |
| *Clinopodium vulgare* L. | MIB:ZPL:03663 | Common | HE963423 | HE966576 |
| *Colchicum autumnale* L. | MIB:ZPL:01701 | Common | HF572820 | FR865109 |
| *Commelina communis* L. | MIB:ZPL:03251 | Alien | HE963424 | HE966577 |
| *Consolida ajacis* (L.) Schur | Genbank | Common | FJ626582* | AF216578* |
| *Consolida regalis* Gray | MIB:ZPL:03396 | Common | HE963425 | HE966578 |
| *Convallaria majalis* L. | MIB:ZPL:01662 | Common | HF572828 | FN675807 |
| *Convolvulus arvensis* L. | MIB:ZPL:03389 | Common | HE963426 | HE966579 |
| *Conyza canadensis* (L.) Cronquist. | Genbank | Alien | HQ590045* | HQ596652* |
| *Cornus mas* L. | GenBank | Common | L11216* | JF321221* |
| *Cornus sanguinea* L. | MIB:ZPL:03130 | Common | HE963427 | HE966580 |
| *Corylus avellana* L. | MIB:ZPL:02028 | Common | FR865138 | FR865092 |
| *Cotinus coggygria* Scop. | MIB:ZPL:03047 | Common | HE963431 | HE966584 |
| *Cotoneaster integerrimus* Medik. | Genbank | Common | JQ391316* | JQ390682* |
| *Crataegus monogyna* Jacq. | MIB:ZPL:03041 | Common | HE963432 | HE966585 |
| *Crepis aurea* (L.) Cass. | Genbank | Rare | JN892650* | JX501945* |
| *Crepis capillaris* (L.) Wallr. | Genbank | Common | HM849923* | FJ395464* |
| *Crepis setosa* Haller | MIB:ZPL:03394 | Common | HE963436 | HE966589 |
| *Cupressus sempervirens* L. | MIB:ZPL:03542 | Alien | HE963440 | HE966593 |
| *Cyanus triumfettii* (All.) Dostál ex Á. & D. Löve | MIB:ZPL:03492 | Common | HG416987 | HG800517 |
| *Cynodon dactylon* (L.) Pers. | MIB:ZPL:03570 | Common | HE963441 | HE966594 |
| *Cystopteris alpina* (Lam.) Desv. | Genbank | Common | JX874043* | HQ157289* |
| *Cytisus nigricans* L. | MIB:ZPL:03055 | Common | HE963443 | HE966595 |
| *Dactylis glomerata* L. | MIB:ZPL:03069 | Common | HE963444 | HE966596 |
| *Dactylorhiza maculata* (L.) Soó | MIB:ZPL:03457 | Common | HG416988 | HG800518 |
| *Daphne laureola* L. | GenBank | Common | JN892068* | GQ167448* |
| *Daphne mezereum* L. | GenBank | Common | JN892066* | GQ167487* |
| *Daphne striata* Tratt. | MIB:ZPL:07311 | Rare | HG416989 | HG800519 |
| *Datura stramonium*L. | MIB:ZPL:03684 | Alien | HE963445 | HE966597 |
| *Daucus carota* L. | MIB:ZPL:04001 | Common | HE963446 | HE966598 |
| *Deschampsia cespitosa* (L.) P. Beauv | Genbank | Common | EF125152* | FJ395500* |
| *Dianthus monspessulanus* L. | MIB:ZPL:03659 | Common | HE963447 | HE966599 |
| *Dianthus sylvestris* Wulfen | MIB:ZPL:03454 | Common | HG416990 | HG800520 |
| *Digitalis lutea* L. | MIB:ZPL:03473 | Common | HG416991 | HG800521 |
| *Digitaria sanguinalis* (L.) Scop. | Genbank | Alien | HQ590067* | HQ596673* |
| *Diospyros lotus* L. | MIB:ZPL:03689 | Alien | HE963452 | HE966604 |
| *Diphasiastrum complanatum* (L.) Holub | Genbank | Common | AB574627* | AB575304* |
| *Dipsacus fullonum* L. | Genbank | Alien | HQ590068* | HQ596674* |
| *Doronicum pardalianches* L. | Genbank | Common | GU817757* | EF538088* |
| *Dorycnium pentaphyllum* Scop. | MIB:ZPL:01714 | Common | FR865124 | FR865078 |
| *Dryopteris filix-mas* (L.) Schott | GenBank | Common | HE574628* | JN189398* |
| *Echinochloa crus-galli* (L.) P. Beauv. | Genbank | Common | KC164307* | HQ600068* |
| *Echium vulgare* L. | MIB:ZPL:03538 | Common | HE963457 | HE966609 |
| *Eleocharis palustris* (L.) Roem. & Schult. *palustris* | GenBank | Common | KC584890* | KC584961* |
| *Eleusine indica* (L.) Gaertn. | Genbank | Alien | EF125108* | HQ600074* |
| *Elodea canadensis* Michx. | Genbank | Alien | JX100687* | JX100539* |
| *Elymus repens* (L.) Gould | Genbank | Common | HQ590076* | HQ596680* |
| *Emerus major* Mill. | MIB:ZPL:03261 | Common | HE963459 | HE966611 |
| *Empetrum nigrum* L. | Genbank | Common | AF419822* | GU361912* |
| *Epilobium angustifolium*L. | GenBank | Common | JN965439* | JN044476* |
| *Epilobium palustre* L. | Genbank | Common | JF941483* | JN044479* |
| *Epipactis atrorubens* (Hoffm. ex Bernh.) Besser | GenBank | Common | JN891058* | JN847386* |
| *Epipactis helleborine* (L.) Crantz | Genbank | Rare | HQ700738* | HQ596681* |
| *Equisetum arvense* L. | GenBank | Common | HQ590081* | HQ596682* |
| *Equisetum hyemale* L. | Genbank | Common | EU677110* | EU750649* |
| *Equisetum palustre* L. | Genbank | Common | AB574686* | AB575349* |
| *Equisetum ramosissimum* Desf. | Genbank | Common | AB574688* | AB575350* |
| *Equisetum telmateia* Ehrh. | Genbank | Common | AF313580* | GQ248298* |
| *Equisetum variegatum* Schleich. ex Weber & D. Mohr | Genbank | Common | AY226134* | AB575353* |
| *Eragrostis cilianensis* (All.) Vignolo Lutati ex Janch. | Genbank | Common | FN870390* | HQ876964* |
| *Eragrostis pilosa* (L.) P. Beauv. | MIB:ZPL:03980 | Common | HE963460 | HE966612 |
| *Erica carnea* L. | MIB:ZPL:04506 | Common | HE963461 | HE966613 |
| *Erigeron annuus* (L.) Desf. | MIB:ZPL:03187 | Common | HE963464 | HE966616 |
| *Eriobotrya japonica* (Thunb.) Lindl. | Genbank | Alien | JQ391272* | GQ305326* |
| *Erophila verna* (L.) DC. | Genbank | Common | HQ619740* | FJ493271* |
| *Eryngium campestre* L. | Genbank | Common | JN892017* | HE602478* |
| *Euonymus europaeus*L. | MIB:ZPL:03248 | Common | HE963469 | HE966621 |
| *Euphorbia amygdaloides* L. | MIB:ZPL:03426 | Common | HG416992 | HG800522 |
| *Euphorbia cyparissias* L. | MIB:ZPL:03056 | Common | HE963476 | HE966627 |
| *Euphorbia dulcis* L. | MIB:ZPL:03471 | Common | HG416993 | HG800523 |
| *Euphorbia helioscopia* L. | MIB:ZPL:03203 | Common | HE963477 | HE966628 |
| *Euphorbia lathyris* L. | Genbank | Common | HM849989* | GQ434952* |
| *Euphorbia peplus* L. | MIB:ZPL:03210 | Common | HE963480 | HE966631 |
| *Euphorbia variabilis* Ces. | MIB:ZPL:07312 | Rare | HG416994 | HG800524 |
| *Fagopyrum esculentum* Moench. | Genbank | Alien | EF653762* | EF653736* |
| *Fagus sylvatica* L. | GenBank | Common | JN891396* | FN687511* |
| *Fallopia convolvulus* (L.) Á. Löve | MIB:ZPL:04515 | Common | HE963483 | HE966634 |
| *Festuca arundinacea* Schreb. | Genbank | Common | HQ590163* | HQ596753* |
| *Festuca heterophylla* Lam. | MIB:ZPL:03058 | Common | HE963485 | HE966635 |
| *Festuca ovina* L. | Genbank | Common | HQ600423* | HQ600116* |
| *Festuca rubra* L. | MIB:ZPL:03213 | Common | HE963486 | HE966636 |
| *Ficus carica* L. | MIB:ZPL:03207 | Alien | HE963487 | HE966637 |
| *Foeniculum vulgare* Mill. | Genbank | Common | GQ120445* | AY587874* |
| *Fragaria chiloensis* (L.) Duchesne | Genbank | Common | JX402830* | GQ476754* |
| *Fragaria moschata* Duchesne | GenBank | Common | JX469002* | GQ476764* |
| *Fragaria vesca* L. | GenBank | Common | JN892328* | FJ395465* |
| *Frangula alnus* Miller | GenBank | Common | JN892060* | EU750523* |
| *Fraxinus excelsior* L. | GenBank | Common | JN891186* | HM367417* |
| *Fraxinus ornus* L. *ornus* | MIB:ZPL:03045 | Common | HE963491 | HE966641 |
| *Gagea lutea* (L.) Ker Gawl. | GenBank | Common | JN891145* | EU939250* |
| *Galinsoga parviflora* Cav. | Genbank | Alien | DQ006065* | DQ006151* |
| *Galium aparine* L. | MIB:ZPL:03129 | Common | HE963496 | HE966646 |
| *Galium lucidum* All. | MIB:ZPL:03111 | Common | HE963497 | HE966647 |
| *Galium mollugo* L. | GenBank | Common | JN893407* | HQ596710* |
| *Galium palustre* L. | Genbank | Common | HQ590113* | HQ596711* |
| *Galium verum* L. | GenBank | Common | JN892891* | HQ596713* |
| *Genista radiata* (L.) Scop. | MIB:ZPL:04263 | Common | HG416995 | HG800525 |
| *Genista tinctoria* L. | MIB:ZPL:03064 | Common | HE963498 | HE966648 |
| *Geranium columbinum* L. | MIB:ZPL:03081 | Common | HE963499 | HE966649 |
| *Geranium molle* L. | MIB:ZPL:04512 | Common | HE963500 | HE966650 |
| *Geranium nodosum* L. | MIB:ZPL:03523 | Common | HG416996 | HG800526 |
| *Geranium phaeum* L. | MIB:ZPL:03470 | Common | HG416997 | HG800527 |
| *Geranium robertianum* L. | MIB:ZPL:03464 | Common | HG416998 | HG800528 |
| *Geranium rotundifolium* L. | MIB:ZPL:04329 | Common | HG416999 | HG800529 |
| *Geranium sanguineum* L. | MIB:ZPL:03428 | Common | HG417000 | HG800530 |
| *Geranium sylvaticum* L. | MIB:ZPL:03469 | Common | HG417001 | HG800531 |
| *Geum urbanum* L. | MIB:ZPL:03128 | Common | HE963503 | HE966653 |
| *Glechoma hederacea* L. | GenBank | Common | JN890905* | DQ667355* |
| *Globularia cordifolia* L. | MIB:ZPL:01709 | Common | FR865123 | FR865077 |
| *Glyceria fluitans* (L.) R. Br. | Genbank | Common | JN891742* | DQ665506* |
| *Glyceria notata* Chevall. | Genbank | Common | JN892963* | DQ665519* |
| *Goodyera repens* (L.) R. Br. | Genbank | Common | KC704898* | HM021604* |
| *Gymnadenia rhellicani* (Teppner & E. Klein) Teppner & E. Klein | MIB:ZPL:04331 | Common | HG417016 | HG800546 |
| *Gymnocarpium dryopteris* (L.) Newman | GenBank | Common | KF186524* | HQ596723* |
| *Gypsophila repens* L. | MIB:ZPL:04315 | Common | HG417002 | HG800532 |
| *Hedera helix* L. | MIB:ZPL:03037 | Alien | HE963508 | HE966658 |
| *Helianthemum nummularium* (L.) Mill. | MIB:ZPL:03113 | Common | HE963509 | HE966659 |
| *Helianthus annuus* L. | Genbank | Alien | L13929* | JX912584* |
| *Helianthus tuberosus* L. | Genbank | Alien | HQ219825* | JX912643* |
| *Heliotropium europaeum* L. | Genbank | Common | HM850049* | HQ286215* |
| *Helleborus viridis* L. | GenBank | Common | JN893217* | FJ493302* |
| *Hemerocallis fulva* (L.) L. | Genbank | Alien | KC704806* | KC704274* |
| *Heracleum sphondylium* L. | GenBank | Common | JN892744* | EU594928* |
| *Hesperis matronalis* L. | GenBank | Common | HQ590129* | HQ596725* |
| *Heteropogon contortus* (L.) P. Beauv. | Genbank | Common | AM235061* | HQ876977* |
| *Hibiscus syriacus* L. | MIB:ZPL:04011 | Alien | HE963510 | HE966660 |
| *Hieracium pilosella* L. | MIB:ZPL:03084 | Common | HE963511 | HE966661 |
| *Hieracium piloselloides* Vill. | MIB:ZPL:03245 | Common | HE963512 | HE966662 |
| *Hippocrepis comosa* L. | MIB:ZPL:03491 | Common | HG417003 | HG800533 |
| *Holcus lanatus* L. | Genbank | Common | JN892327* | HQ600148* |
| *Hordeum murinum* L. | MIB:ZPL:03078 | Common | HE963513 | HE966663 |
| *Hordeum vulgare* L. | Genbank | Alien | AY137453* | HQ600125* |
| *Horminum pyrenaicum* L. | MIB:ZPL:03490 | Common | HG417004 | HG800534 |
| *Humulus lupulus* L. | Genbank | Common | DQ006077* | DQ006164* |
| *Huperzia selago* (L.) Bernh. ex Schrank & Mart. | Genbank | Common | AB574636* | AB575312* |
| *Hylotelephium telephium* (L.) H.Ohba | GenBank | Common | HQ590138* | HQ596731* |
| *Hyoscyamus albus* L. | Genbank | Common | HQ216122* | HQ216158* |
| *Hyoscyamus niger L.* | Genbank | Common | HQ216125* | HQ216162* |
| *Hypericum androsaemum* L. | Genbank | Common | JN892756* | KC709196* |
| *Hypericum hirsutum* L. | Genbank | Common | JN892839* | KC709195* |
| *Hypericum humifusum* L. | Genbank | Common | HM850063* | FJ788903* |
| *Hypericum maculatum* Crantz | GenBank | Common | JN892622* | KC709213* |
| *Hypericum montanum* L. | GenBank | Common | JN892623* | KC709270* |
| *Hypericum perforatum* L. | MIB:ZPL:03678 | Common | HE963515 | HE966665 |
| *Hypochaeris radicata* L. | GenBank | Common | JN892418* | FJ395488* |
| *Ilex aquifolium* L. | MIB:ZPL:04014 | Common | HE963516 | HE966666 |
| *Inula britannica* L. | Genbank | Common | GU724234* | GU724259* |
| *Inula helenium* L. | Genbank | Common | HQ590141* | HQ596734* |
| *Inula hirta* L. | MIB:ZPL:03067 | Common | HE963517 | HE966667 |
| *Inula salicina* L. | MIB:ZPL:01672 | Common | FR865116 | FR865070 |
| *Iris germanica* L. | Genbank | Alien | JF942051* | JN044979* |
| *Iris pseudacorus* L. | Genbank | Common | KC584881* | KC584954* |
| *Juglans regia* L. | MIB:ZPL:03202 | Common | HE963521 | HE966671 |
| *Juncus effusus* L. | Genbank | Common | HQ590146* | HQ596739* |
| *Juniperus communis* L. | MIB:ZPL:03400 | Common | HE963522 | HE966672 |
| *Knautia arvensis* (L.) Coult. | MIB:ZPL:03422 | Common | HG417005 | HG800535 |
| *Koeleria lobata* (M.Bieb.) Roem. & Schult.. | MIB:ZPL:03228 | Common | HE963525 | HE966675 |
| *Laburnum anagyroides* Medik. | MIB:ZPL:03455 | Common | HG417006 | HG800536 |
| *Lactuca muralis* (L.)Gaertn. | MIB:ZPL:03687 | Common | HE963528 | HE966677 |
| *Lactuca serriola* L. | MIB:ZPL:03561 | Common | HE963529 | HE966678 |
| *Lamium album* L. | GenBank | Common | JN891647* | FJ395503* |
| *Lamium amplexicaule* L. | Genbank | Common | HQ902760* | HQ902820* |
| *Lamium galeobdolon* L. | GenBank | Common | JN893549* | JF780145* |
| *Lamium maculatum* L. | MIB:ZPL:03189 | Common | HE963530 | HE966679 |
| *Lamium purpureum* L. | GenBank | Common | JN891285* | FJ493284* |
| *Lapsana communis* L. | Genbank | Common | HM850099* | FJ395498* |
| *Larix decidua* Mill. | GenBank | Common | FN689379* | FN689386* |
| *Laserpitium nitidum* Zanted. | MIB:ZPL:07313 | Common | HG417007 | HG800537 |
| *Lathyrus aphaca* L. | Genbank | Common | JX505474* | JX505919* |
| *Lathyrus laevigatus* (Waldst. & Kit.) Gren. | GenBank | Common | JX505481* | JX505932* |
| *Lathyrus niger* (L.) Bernh. | MIB:ZPL:03044 | Common | HE963532 | HE966681 |
| *Lathyrus pratensis* L. | MIB:ZPL:03226 | Common | HE963535 | HE966684 |
| *Lathyrus sylvestris* L. *sylvestris* | Genbank | Common | JN891874* | JQ886555* |
| *Laurus nobilis* L. | MIB:ZPL:03036 | Common | HE963536 | HE966685 |
| *Lavandula angustifolia* Mill. *angustifolia* | MIB:ZPL:03990 | Common | HE963537 | HE966686 |
| *Lemna minor* L. | Genbank | Common | HQ590155* | HQ596744* |
| *Leontodon hispidus* L. | MIB:ZPL:02803 | Common | FR865147 | FR865098 |
| *Leontopodium alpinum* Cass. | MIB:ZPL:07314 | Common | HG417008 | HG800538 |
| *Leonurus cardiaca* L. *cardiaca* | Genbank | Common | HQ839686* | FJ513116* |
| *Leopoldia comosa* (L.) Parl. | MIB:ZPL:06932 | Common | HF572827 | HF572805 |
| *Lepidium draba* L. *draba* | MIB:ZPL:03234 | Common | HE963538 | HE966687 |
| *Lepidium ruderale* L. | Genbank | Common | JF942219* | JN045148* |
| *Leucanthemum vulgare* Lam. | GenBank | Common | JN893039* | HQ596748* |
| *Ligustrum lucidum* Aiton | Genbank | Common | JF942289* | JN045229* |
| *Ligustrum vulgare* L. | MIB:ZPL:03079 | Common | HE963539 | HE966688 |
| *Lilium bulbiferum* L. *croceum* (Chaix) Jan | MIB:ZPL:03451 | Common | HG417009 | HG800539 |
| *Lilium candidum* L. | Genbank | Common | AB034928* | AJ431692* |
| *Limodorum abortivum* (L.) Sw. | MIB:ZPL:03452 | Common | HG417010 | HG800540 |
| *Linaria vulgaris* Mill. | Genbank | Common | HQ590160* | HQ596749* |
| *Linum alpinum* Jacq. | MIB:ZPL:07315 | Common | HG417011 | HG800541 |
| *Linum flavum* L. | Genbank | Common | HM544065* | GQ845282* |
| *Linum tenuifolium* L. | MIB:ZPL:03235 | Common | HE963540 | HE966689 |
| *Linum usitatissimum* L. | Genbank | Common | JX664057* | GQ845304* |
| *Liriodendron tulipifera* L. | MIB:ZPL:03982 | Common | HE963541 | HE966690 |
| *Lithospermum officinale* L. | Genbank | Common | JN893110* | FJ827351* |
| *Lolium multiflorum* Lam. | Genbank | Common | FN870397* | HQ600142* |
| *Lolium perenne* L. | MIB:ZPL:03214 | Common | HE963542 | HE966691 |
| *Lonicera japonica* Thunb. | MIB:ZPL:03571 | Alien | HE963545 | HE966694 |
| *Lotus corniculatus* L. | MIB:ZPL:03480 | Common | HG417012 | HG800542 |
| *Lunaria annua* L. | MIB:ZPL:03549 | Common | HE963547 | HE966696 |
| *Luzula campestris* (L.) DC. | Genbank | Common | JN893077* | FJ395506* |
| *Lycopodium annotinum* L. | Genbank | Common | AB574622* | AB575301* |
| *Lycopodium clavatum* L. | Genbank | Common | AB574626* | AB575303* |
| *Lythrum salicaria* L. | Genbank | Common | JN893193* | HQ596761* |
| *Maianthemum bifolium* (L.) Schmidt | MIB:ZPL:03449 | Common | HG417013 | HG800543 |
| *Malus pumila* Mill. | MIB:ZPL:03405 | Common | HE963549 | HE966698 |
| *Malva alcea* L. | Genbank | Common | GQ248640* | GQ248337* |
| *Malva neglecta* Wallr. | Genbank | Common | HQ590176* | HQ596765* |
| *Malva parviflora* L. | Genbank | Common | JQ412388* | EF419604* |
| *Malva sylvestris* L. | MIB:ZPL:03184 | Common | HE963550 | HE966699 |
| *Malva verticillata* L. | Genbank | Alien | JQ933397* | EF419605* |
| *Marrubium vulgare* L. | Genbank | Common | HM590056* | HM590121* |
| *Matricaria chamomilla* L. | Genbank | Common | JN893454* | EU547790* |
| *Medicago lupulina* L. | MIB:ZPL:03072 | Common | HE963556 | HE966705 |
| *Medicago minima* (L.) L. | MIB:ZPL:03193 | Common | HE963557 | HE966706 |
| *Medicago sativa* L. | MIB:ZPL:03404 | Alien | HE963558 | HE966707 |
| *Melica nutans* L. | GenBank | Common | HQ600444* | HQ600137* |
| *Melica uniflora* Retz. | Genbank | Common | JN893344* | FJ395479* |
| *Melilotus albus* Medik. | MIB:ZPL:03563 | Common | HE963560 | HE966709 |
| *Melilotus officinalis* (L.) Pall. | MIB:ZPL:03401 | Common | HE963561 | HE966710 |
| *Melissa officinalis* L. | MIB:ZPL:03435 | Common | HF565314 | HF565305 |
| *Melittis melissophyllum*L. | MIB:ZPL:03514 | Common | HG417014 | HG800544 |
| *Mentha aquatica* L. | MIB:ZPL:03782 | Common | FR720533 | FR726100 |
| *Mentha arvensis*L. | Genbank | Common | HQ590183* | HQ596770* |
| *Mentha longifolia* (L.) Huds. | MIB:ZPL:03237 | Common | HE963563 | HE966712 |
| *Mentha pulegium* L. | MIB:ZPL:03125 | Common | HE963564 | HE966713 |
| *Mercurialis annua* L. | MIB:ZPL:03677 | Common | HE963565 | HE966714 |
| *Mercurialis perennis* L. | MIB:ZPL:03979 | Common | FR865155 | FR865106 |
| *Milium effusum* L. | Genbank | Common | JN893348* | HQ600161* |
| *Minuartia grignensis* (Rchb.) Mattf. | MIB:ZPL:07316 | Rare | HG417015 | HG800545 |
| *Misopates orontium* (L.) Raf. | Genbank | Common | HM850180* | HM152879* |
| *Morus alba* L. | Genbank | Alien | KC584883* | KC584956* |
| *Myriophyllum spicatum* L. | Genbank | Common | GU135245* | GU135416* |
| *Myrtus communis* L. | MIB:ZPL:03543 | Common | HE963567 | HE966716 |
| *Najas marina* L. | Genbank | Common | HM240504* | HQ687183* |
| *Narcissus tazetta* L. | Genbank | Rare | GQ436660* | GQ435346* |
| *Nardus stricta* L. | Genbank | Common | JN893338* | EU489285* |
| *Nasturtium officinale* R. Br. | Genbank | Common | HQ590244* | HQ596821* |
| *Nepeta cataria* L. | Genbank | Common | JX520953* | DQ667388* |
| *Nerium oleander* L. | MIB:ZPL:03675 | Common | HE963568 | HE966717 |
| *Neslia paniculata* (L.) Desv. | Genbank | Common | DQ310541* | FR822354* |
| *Noccaea rotundifolia* (L.) Moench *grignensis* F.K. Mey. | MIB:ZPL:07321 | Rare | HG417053 | HG800583 |
| *Ocimum basilicum* L. | MIB:ZPL:02996 | Alien | FR720554 | FR726121 |
| *Oenothera biennis* L. | Genbank | Alien | DQ006109* | DQ006206* |
| *Olea europaea* L. | MIB:ZPL:03402 | Common | HE963570 | HE966718 |
| *Ononis natrix* L. | Genbank | Common | JQ858242* | EU531707* |
| *Ononis spinosa* L. | MIB:ZPL:03664 | Common | HE963572 | HE966720 |
| *Ophioglossum vulgatum* L. | Genbank | Common | AB574681* | AB575344* |
| *Ophrys apifera* Huds. | Genbank | Rare | JN890698* | AM711642* |
| *Ophrys fuciflora* (F.W. Schmidt) Moench | Genbank | Rare | GQ248658* | GQ248355* |
| *Ophrys insectifera* L. | Genbank | Rare | HE858481* | AM711676* |
| *Orchis mascula* (L.) L. | MIB:ZPL:04267 | Common | HG417017 | HG800547 |
| *Origanum majorana* L. | MIB:ZPL:03791 | Common | FR720558 | FR726125 |
| *Origanum vulgare* L. | MIB:ZPL:01721 | Common | FR865126 | FR865080 |
| *Ostrya carpinifolia* Scop. | MIB:ZPL:03539 | Common | HE963575 | HE966721 |
| *Oxalis acetosella* L. | GenBank | Common | JN893025* | JN592057* |
| *Oxalis corniculata* L. | MIB:ZPL:03190 | Common | HE963577 | HE966723 |
| *Paliurus spina-christi* Mill. | Genbank | Common | AJ390051* | EU075112* |
| *Panicum miliaceum* L. | Genbank | Alien | FR667683* | FR667854* |
| *Papaver rhoeas* L. | MIB:ZPL:03217 | Alien | HE963579 | HE966724 |
| *Papaver somniferum* L. | Genbank | Common | JN114830* | JN584668* |
| *Parietaria officinalis* L. | MIB:ZPL:03134 | Common | HE963580 | HE966725 |
| *Paris quadrifolia* L. | MIB:ZPL:03439 | Common | HG417018 | HG800548 |
| *Parnassia palustris* L. | MIB:ZPL:04298 | Common | HG417019 | HG800549 |
| *Parthenocissus quinquefolia* (L.) Planch. | MIB:ZPL:03135 | Alien | HE963582 | HE966726 |
| *Passiflora coerulea* L. | Genbank | Common | HM850239* | AY032816* |
| *Paulownia tomentosa* (Thunb.) Steud. | Genbank | Alien | L36447* | JF321288* |
| *Pedicularis gyroflexa* Vill. | MIB:ZPL:04278 | Rare | HG417020 | HG800550 |
| *Pelargonium × hortorum* L.H. Bailey | MIB:ZPL:07365 | Alien | HG800593 | HG800592 |
| *Persicaria lapathifolia* (L.) Delarbre | MIB:ZPL:03999 | Common | HE963584 | HE966728 |
| *Persicaria orientalis* (L.) Spach | Genbank | Alien | EU554013* | FJ503035* |
| *Petrorhagia saxifraga* (L.) Link | MIB:ZPL:03231 | Common | HE963585 | HE966729 |
| *Petroselinum crispum* (Mill.) Fuss | MIB:ZPL:04520 | Alien | HF565315 | HF565306 |
| *Petunia hybrida* Vilm. | Genbank | Common | HM850249* | JX856336* |
| *Phalaris arundinacea* L. | GenBank | Common | HQ600446* | HQ600139* |
| *Phaseolus coccineus* L. | Genbank | Common | GQ411655* | FJ951185* |
| *Phaseolus vulgaris* L. | Genbank | Common | GQ411631* | FJ951239* |
| *Phegopteris connectilis* (Michx.) Watt | GenBank | Common | KF186501* | HQ890382* |
| *Phleum pratense* L. | Genbank | Common | HQ590204* | HQ596784* |
| *Phragmites australis* (Cav.) Trin. ex Steud. | Genbank | Common | HQ590205* | HQ596785* |
| *Phyllostachys aurea* (Carrière) A.Rivière & C.Rivière | Genbank | Alien | GU135227* | GU135398* |
| *Physalis alkekengi* L. | Genbank | Common | GQ436615* | GQ435282* |
| *Physoplexis comosa* (L.) Schur | MIB:ZPL:07317 | Rare | HG417021 | HG800551 |
| *Phyteuma betonicifolium*Vill. | MIB:ZPL:03442 | Rare | HG417022 | HG800552 |
| *Phyteuma ovatum* Honck. | MIB:ZPL:03524 | Common | HG417023 | HG800553 |
| *Phyteuma scheuchzeri* All. | MIB:ZPL:03505 | Common | HG417024 | HG800554 |
| *Phytolacca americana* L. | MIB:ZPL:04009 | Alien | HE963589 | HE966733 |
| *Picea abies* (L.) H. Karst. | MIB:ZPL:04010 | Rare | HE963590 | HE966734 |
| *Picris hieracioides* L. | MIB:ZPL:03376 | Common | HE963593 | HE966737 |
| *Pimpinella saxifraga* L. | MIB:ZPL:02013 | Common | FR865128 | FR865082 |
| *Pinus mugo* Turra *uncinata* (Ramond ex DC.)  Domin | Genbank | Common | FR831923* | FR832532* |
| *Pinus mugo* Turro | GenBank | Common | FR831919* | FR832535* |
| *Pinus strobus* L. | Genbank | Alien | EU677090* | EU750631* |
| *Pinus sylvestris* L. | Genbank | Common | EU677093* | EU750634* |
| *Pisum sativum* L. | Genbank | Alien | JN661190* | JX505967* |
| *Plantago lanceolata* L. | MIB:ZPL:03104 | Common | HE963598 | HE966742 |
| *Plantago major* | MIB:ZPL:03127 | Common | HE963599 | HE966743 |
| *Platanthera bifolia* (L.) Rchb. | MIB:ZPL:03458 | Common | HG417025 | HG800555 |
| *Platanus orientalis* L. | Genbank | Alien | JN114832* | HE661222* |
| *Poa alpina* L. | Genbank | Common | KC483484* | KC476111* |
| *Poa annua* L. | MIB:ZPL:03262 | Common | HE963600 | HE966744 |
| *Poa bulbosa* L. | MIB:ZPL:03054 | Common | HE963601 | HE966745 |
| *Poa compressa* L. | Genbank | Common | EU676942* | EU750483* |
| *Poa pratensis* L. | Genbank | Common | HQ590213* | HQ596794* |
| *Poa trivialis* L. | Genbank | Common | HM850270* | FJ395457* |
| *Polycarpon tetraphyllum* (L.) L. | MIB:ZPL:03546 | Common | HE963603 | HE966747 |
| *Polygala chamaebuxus* L. | MIB:ZPL:03456 | Common | HG417026 | HG800556 |
| *Polygala nicaeensis* W.D.J. Koch *carniolica* (A. Kern.) P. Graebn. | MIB:ZPL:03495 | Common | HG417027 | HG800557 |
| *Polygonatum odoratum* (Mill.) Druce | GenBank | Common | JN890573* | GQ434925* |
| Polygonatum verticillatum (L.) All. | GenBank | Common | JF943498* | JN046422* |
| *Polygonum aviculare* L. | Genbank | Common | EU676946* | EU750487* |
| *Polypodium vulgare* L. | GenBank | Common | HE574634* | HQ15729* |
| *Polystichum braunii* (Spenn.) Fée | Genbank | Common | AB575187* | AB575806* |
| *Populus alba* L. | Genbank | Common | HM850277* | JF429903* |
| *Populus deltoides* Marshall | Genbank | Alien | EU676958* | EU750499* |
| *Populus nigra* L. | MIB:ZPL:03257 | Common | HE963608 | HE966752 |
| *Populus tremula* L. | MIB:ZPL:03536 | Common | HE963609 | HE966753 |
| *Portulaca oleracea* L. | MIB:ZPL:03669 | Common | HE963610 | HE966754 |
| *Potamogeton crispus* L. | Genbank | Common | JF943539* | DQ786528* |
| *Potamogeton perfoliatus* L. | Genbank | Common | JF943569* | DQ786545* |
| *Potamogeton pusillus* L. | Genbank | Common | JF943574* | DQ786548* |
| *Potentilla aurea* L. | MIB:ZPL:03460 | Common | HG417028 | HG800558 |
| *Potentilla caulescens* L. | MIB:ZPL:04275 | Common | HG417029 | HG800559 |
| *Potentilla crantzii* (Crantz) Beck ex Fritsch | GenBank | Common | JN893502* | JX276840* |
| *Potentilla erecta* (L.) Raeusch. | GenBank | Common | JN893501* | GQ384959* |
| *Potentilla indica* (Jacks.) Th. Wolf | MIB:ZPL:04517 | Common | HE963611 | HE966755 |
| *Potentilla nitida* L. | MIB:ZPL:07318 | Common | HG417030 | HG800560 |
| *Potentilla recta* L. | GenBank | Common | HQ590222* | GQ384960* |
| *Potentilla reptans* L. | MIB:ZPL:03532 | Common | HE963612 | HE966756 |
| *Primula glaucescens* Moretti | MIB:ZPL:04304 | Rare | HG417031 | HG800561 |
| *Primula grignensis* Moser | MIB:ZPL:07319 | Rare | HG417032 | HG800562 |
| *Primula veris* L. | GenBank | Common | JN893423* | JX231087* |
| *Primula vulgaris* Huds. | Genbank | Common | JN892823* | FJ493301* |
| *Prunella grandiflora* (L.) Scholler | MIB:ZPL:04260 | Common | HG417033 | HG800563 |
| *Prunella vulgaris* L. | MIB:ZPL:03431 | Common | HG417034 | HG800564 |
| *Prunus avium* L. | MIB:ZPL:03132 | Common | HE963614 | HE966758 |
| *Prunus cerasus*L. | Genbank/MIB:ZPL:01684 | Common | JN893012* | FN675832 |
| *Prunus domestica* L. | MIB:ZPL:03551 | Alien | HE963617 | HE966761 |
| *Prunus dulcis* (Mill.) D.A. Webb | Genbank | Common | HQ235365* | HQ188715* |
| *Prunus laurocerasus* L. | MIB:ZPL:03558 | Common | HE963618 | HE966762 |
| *Prunus mahaleb* L. | MIB:ZPL:03109 | Common | HE963619 | HE966763 |
| *Prunus persica* (L.) Batsch | MIB:ZPL:03259 | Common | HE963620 | HE966764 |
| *Prunus spinosa* L. | MIB:ZPL:03120 | Common | HE963621 | HE966765 |
| *Pseudolysimachion spicatum* (L.) Opiz | GenBank | Common | JN892244* | FJ848104* |
| *Pteridium aquilinum* (L.) Kuhn | MIB:ZPL:02811 | Common | FR865150 | FR865101 |
| *Pteris cretica* L. | Genbank | Common | AB574825* | AB575477* |
| *Pulicaria dysenterica* (L.) Bernh. | Genbank | Common | JN892485* | FM998693* |
| *Pulmonaria officinalis* L. | GenBank | Common | HQ619735* | JX196825* |
| *Punica granatum* L. | MIB:ZPL:03531 | Common | HE963623 | HE966767 |
| *Quercus cerris* L. | Genbank | Common | FN675724* | FN687488* |
| *Quercus ilex* L. | MIB:ZPL:03105 | Common | HE963625 | HE966769 |
| *Quercus petraea* (Mattuschka) Liebl. | MIB:ZPL:03049 | Common | HE963626 | HE966770 |
| Quercus pubescens Willd. | MIB:ZPL:03050 | Common | HE963627 | HE966771 |
| *Quercus robur* L. | Genbank | Common | FN675735* | FN687492* |
| *Quercus suber* L. | Genbank | Common | FN675728* | HE591255* |
| *Ranunculus acris* L. | GenBank | Common | JN891741* | FJ395535* |
| *Ranunculus bulbosus* L. | MIB:ZPL:03074 | Common | HE963628 | HE966772 |
| *Ranunculus repens* L. | Genbank | Common | JN893674* | FJ395466* |
| *Raphanus raphanistrum* L. | Genbank | Common | EF590570* | GQ248383* |
| *Rhamnus cathartica* L. | Genbank | Common | EU676980* | EU750521* |
| *Rhinanthus alectorolophus* (Scop.) Pollich | MIB:ZPL:03481 | Common | HG417035 | HG800565 |
| *Rhododendron ferrugineum* L. | MIB:ZPL:02916 | Common | HE585265 | HE585250 |
| *Rhododendron hirsutum* L. | MIB:ZPL:04268 | Common | HG417036 | HG800566 |
| *Ribes rubrum* L. | Genbank | Common | HQ590241* | HQ596818* |
| *Ricinus communis* L. | Genbank | Common | GU135207* | GU135374* |
| *Robinia pseudacacia* L. | MIB:ZPL:03046 | Common | HE963631 | HE966775 |
| *Rosa arvensis* Huds. | Genbank | Common | JN893272* | DQ778748* |
| *Rosa canina* L. | MIB:ZPL:03450 | Common | HG417037 | HG800567 |
| *Rosmarinus officinalis* L. | MIB:ZPL:03403 | Common | HE963635 | HE966779 |
| *Rubus caesius* L. | GenBank | Common | FN689382* | FN687517* |
| *Rubus idaeus* L. | MIB:ZPL:04264 | Common | HG417038 | HG800568 |
| *Rubus ulmifolius* Schott | MIB:ZPL:03221 | Common | HE963637 | HE966782 |
| *Rumex acetosa* L. | GenBank | Common | JN892337* | FJ395473* |
| *Rumex acetosella* L. | GenBank | Common | JN893332* | HQ596827* |
| *Rumex crispus* L. | Genbank | Common | HQ590251* | HQ596828* |
| *Rumex obtusifolius* L. | MIB:ZPL:03186 | Common | HE963640 | HE966785 |
| *Rumex pulcher* L. | MIB:ZPL:03249 | Common | HE963641 | HE966786 |
| *Ruscus aculeatus* L. | MIB:ZPL:03039 | Common | HE963642 | HE966787 |
| *Salix alba* L. | GenBank | Common | FN689366* | FN687514* |
| *Salix aurita* L. | GenBank | Common | JN892179* | GU373277* |
| *Salix babylonica* L. | GenBank | Common | EU676998* | EU750539* |
| *Salix caprea* L. | GenBank | Common | GU373333* | GU373297* |
| *Salix cinerea* L. | Genbank | Common | JN893030* | GU373279* |
| *Salix herbacea* L. | GenBank | Common | GU373338* | GU373281* |
| *Salix myrsinifolia* Salisb. | GenBank | Common | GU373340* | GU373305* |
| *Salix purpurea* L. | GenBank | Common | GU373351* | GU373304* |
| *Salix reticulata* L. | GenBank | Common | JN965972* | GU373293* |
| *Salvia officinalis* L. | MIB:ZPL:03572 | Common | HE963643 | HE966789 |
| *Salvia pratensis* L. | MIB:ZPL:03556 | Common | HE963644 | HE966790 |
| *Salvia verbenaca* L. | GenBank | Common | HQ902795* | HQ902841* |
| *Sambucus ebulus* L. | Genbank/MIB:ZPL:01678 | Common | JN893720* | FN675822 |
| *Sambucus nigra* L. | MIB:ZPL:03430 | Common | HG417039 | HG800569 |
| *Sambucus racemosa* L. | Genbank/MIB:ZPL:01679 | Common | L14066* | FN675823 |
| *Sanguisorba minor* Scop. | MIB:ZPL:03425 | Common | HG417040 | HG800570 |
| *Sanguisorba officinalis* L. | GenBank | Common | JN893531* | GQ435245* |
| *Sanicula europaea* L. | GenBank | Common | JN893724* | FJ395459* |
| *Saponaria officinalis* L. | MIB:ZPL:03384/Genbank | Common | HE963650 | GU440997* |
| *Saussurea alpina* (L.) DC. | GenBank | Common | KC589890* | AY914848* |
| *Saxifraga aizoides* L. | MIB:ZPL:04289 | Common | HG417041 | HG800571 |
| *Saxifraga caesia* L. | MIB:ZPL:04297 | Common | HG417042 | HG800572 |
| *Saxifraga hostii* Tausch | MIB:ZPL:04256 | Common | HG417043 | HG800573 |
| *Saxifraga rotundifolia* L. | MIB:ZPL:04279 | Common | HG417044 | HG800574 |
| *Saxifraga vandellii* Sternb. | MIB:ZPL:07320 | Rare | HG417045 | HG800575 |
| *Scabiosa triandra* L. | MIB:ZPL:03668 | Common | HE963651 | HE966796 |
| *Scirpoides holoschoenus* (L.) Soják | MIB:ZPL:03393 | Common | HE963652 | HE966797 |
| *Scrophularia canina* L. | MIB:ZPL:03378 | Common | HE963653 | HE966798 |
| *Scrophularia nodosa* L. | GenBank | Common | JN892046* | HQ130097* |
| *Secale cereale* L. | GenBank | Common | HQ600458* | HQ600151* |
| *Securigera varia* (L.) Lassen. | MIB:ZPL:03218 | Common | HE963654 | HE966799 |
| *Sedum acre* L. | MIB:ZPL:04319 | Common | HG417046 | HG800576 |
| *Sedum album* L. | MIB:ZPL:04312 | Common | HG417047 | HG800577 |
| *Sedum sexangulare* L. | MIB:ZPL:03242 | Common | HE963657 | HE966802 |
| *Selaginella helvetica* (L.) Spring | GenBank | Common | AB574644* | AB575319* |
| *Senecio jacobaea* L. | MIB:ZPL:03227 | Common | HE963662 | HE966807 |
| *Senecio vulgaris* L. | MIB:ZPL:03216 | Common | HE963663 | HE966808 |
| *Sesleria caerulea* (L.) Ard. | MIB:ZPL:02017 | Common | FR865131 | FR865085 |
| *Setaria italica* (L.) P. Beauv. | GenBank | Common | HQ600402* | HQ600095* |
| *Setaria pumila* (Poir.) Roem. & Schult. | MIB:ZPL:03987 | Common | HE963665 | HE966810 |
| *Setaria verticillata* (L.) P. Beauv. | GenBank | Common | HQ600366* | HQ600059* |
| *Setaria viridis* (L.) P. Beauv. | GenBank | Common | HQ590270* | HQ596844* |
| *Sherardia arvensis* L. | MIB:ZPL:03077 | Common | HE963666 | HE966811 |
| *Silene acaulis* (L.) Jacq | GenBank | Common | JN891508* | AY949842* |
| *Silene dioica* (L.) Clairv. | MIB:ZPL:03521 | Common | HG417048 | HG800578 |
| *Silene latifolia* Poir. *alba* (Mill.) Greuter & Burdet | MIB:ZPL:03082 | Common | HE963669 | HE966814 |
| *Silene nutans* L. | MIB:ZPL:03053 | Common | HE963670 | HE966815 |
| *Silene otites* (L.) Wibel | GenBank | Common | KC171366* | KC211482* |
| *Silene vulgaris* (Moench) Garcke s.l. | MIB:ZPL:03092 | Common | HE963671 | HE966816 |
| *Sinapis arvensis* L. | Genbank | Common | HQ590272* | HQ596845* |
| *Sisymbrium officinale* (L.) Scop. | MIB:ZPL:03204 | Common | HE963679 | HE966824 |
| *Solanum dulcamara* L. | MIB:ZPL:03253 | Common | HE963680 | HE966825 |
| *Solanum lycopersicum* L. | MIB:ZPL:03992 | Alien | HE963681 | HE966826 |
| *Solanum nigrum* L. | MIB:ZPL:04005 | Common | HE963682 | HE966827 |
| *Solanum tuberosum* L. | MIB:ZPL:01694 | Common | HF572814 | FN675829 |
| *Solanum villosum* Mill. | MIB:ZPL:03385 | Common | HE963683 | HE966828 |
| *Solidago canadensis* L. | GenBank | Common | EU677023* | EU750564* |
| *Solidago virgaurea* L. | GenBank | Common | JN893248* | EU337705* |
| *Sonchus arvensis* L. | GenBank | Common | JN893247* | JX501961* |
| *Sonchus asper* (L.) Hill | MIB:ZPL:03110 | Common | HE963686 | HE966831 |
| *Sonchus tenerrimus* L. | GenBank | Common | HM850374* | AY457986* |
| *Sorbus aria* (L.) Crantz | GenBank | Common | JN892380* | GU592794* |
| *Sorbus aucuparia* L. | GenBank | Common | JN893712* | HQ596854* |
| *Sorbus torminalis* (L.) Crantz | MIB:ZPL:03090 | Common | HE963688 | HE966832 |
| *Sorghum halepense* (L.) Pers. | MIB:ZPL:03559/Genbank | Common | HE963689 | HQ600085* |
| *Sporobolus indicus* (L.) R. Br. | GenBank | Common | HE575834* | HQ876999* |
| *Stachys officinalis* (L.) Trevis. | MIB:ZPL:03101 | Common | HE963693 | HE966836 |
| *Stachys sylvatica* L. | GenBank | Common | JN893526* | FJ395546* |
| *Stellaria graminea* L. | GenBank | Common | FJ395572* | FJ395472* |
| *Stellaria holostea* L. | GenBank | Common | FJ395575* | FJ395475* |
| *Syringa vulgaris* L. | MIB:ZPL:03260 | Common | HE963698 | HE966838 |
| *Tamarix gallica* L. | GenBank | Common | KC584887* | KC584958* |
| *Tamus communis* L. | MIB:ZPL:03205 | Common | HE963699 | FR865112 |
| *Tanacetum corymbosum* (L.) Sch. Bip. | MIB:ZPL:03522 | Common | HG417051 | HG800581 |
| *Tanacetum parthenium* (L.) Sch. Bip. | Genbank | Common | HM850390* | AB683415* |
| *Taraxacum officinale* Weber | MIB:ZPL:03126 | Common | HE963702 | HE966842 |
| *Taxus baccata* L. | MIB:ZPL:03035 | Common | HE963703 | HE966843 |
| *Tephroseris integrifolia* (L.) Holub | GenBank | Common | GU817786* | GU818481* |
| *Teucrium chamaedrys* L. | MIB:ZPL:03243 | Common | HE963704 | HE966844 |
| *Teucrium montanum* L. | MIB:ZPL:04288 | Common | HG417052 | HG800582 |
| *Teucrium scordium* L. | GenBank | Common | HQ902769* | HQ902872* |
| *Thelypteris palustris* Schott | GenBank | Common | JN572401* | HQ157288* |
| *Thlaspi arvense* L. | GenBank | Common | HQ590298* | HQ596863* |
| *Thymus praecox* Opiz *polytrichus*(Borbás) Jalas | MIB:ZPL:04846 | Common | HE819509 | HE819473 |
| *Tilia cordata* Mill. | MIB:ZPL:03537 | Common | HE963708 | HE966847 |
| *Tragopogon pratensis* L. | MIB:ZPL:03517 | Common | HG417054 | HG800584 |
| *Traunsteinera globosa* (L.) Rchb. | MIB:ZPL:04317 | Common | HG417055 | HG800585 |
| *Trifolium aureum* Pollich | GenBank | Common | HQ590307* | HQ596870* |
| *Trifolium campestre* Schreb. | MIB:ZPL:03230 | Common | HE963711 | HE966850 |
| *Trifolium montanum* L. | MIB:ZPL:03432 | Common | HG417056 | HG800586 |
| *Trifolium pratense* L. | MIB:ZPL:03208 | Common | HE963713 | HE966852 |
| *Trifolium repens* L. | MIB:ZPL:03232 | Common | HE963714 | HE966853 |
| *Trifolium rubens* L. | MIB:ZPL:03057 | Common | HE963715 | HE966854 |
| *Tripleurospermum inodorum* (L.) Sch. Bip. | Genbank | Common | JN892563* | AB683442* |
| *Tussilago farfara* L. | GenBank | Common | JQ933515* | DQ131857* |
| *Typha latifolia* L. | GenBank | Common | KC584886* | KC584959* |
| *Urtica dioica* L. | MIB:ZPL:03088 | Common | HE963719 | HE966857 |
| *Vaccinium myrtillus* L. | MIB:ZPL:06926 | Common | HF572812 | HF572793 |
| *Vaccinium uliginosum* L. | GenBank | Common | JN966056* | GU361909* |
| *Vaccinium vitis-idaea* L. | GenBank | Common | JN892801* | GU361911* |
| *Valeriana dioica* L. | GenBank | Common | JN892724* | AY794255* |
| *Valeriana montana* L. | GenBank | Common | AY362515* | AY794268* |
| *Valeriana officinalis* L. | GenBank | Common | JN892722* | AY794273* |
| *Valeriana tripteris* L. | GenBank | Common | AF448589* | AY794302* |
| *Valerianella locusta* (L.) Laterr. | GenBank | Common | JN893517* | DQ354227* |
| *Veratrum album* L. | GenBank | Common | D28168* | JF807739* |
| *Veratrum nigrum* L. | Genbank/MIB:ZPL:01720 | Common | GQ436293* | FN675816 |
| *Verbascum chaixii* Vill. | MIB:ZPL:03117 | Common | HE963721 | HE966859 |
| *Verbascum nigrum* L. | MIB:ZPL:04321 | Common | HG417057 | HG800587 |
| *Verbascum phlomoides* L. | MIB:ZPL:03550 | Common | HE963722 | HE966860 |
| *Verbascum thapsus* L. | GenBank | Common | HM850441* | HQ596879* |
| *Verbena officinalis* L. | MIB:ZPL:03545 | Common | HE963723 | HE966861 |
| *Veronica aphylla* L. | MIB:ZPL:07322 | Common | HG417058 | HG800588 |
| *Veronica arvensis* L. | MIB:ZPL:03233 | Common | HE963724 | HE966862 |
| *Veronica beccabunga* L. | GenBank | Common | JN891845* | FJ848099* |
| *Veronica chamaedrys* L. | GenBank | Common | JN891876* | FJ848116* |
| *Veronica officinalis* L. | GenBank | Common | JN893748* | FJ848096* |
| *Veronica persica* Poir. | MIB:ZPL:03121 | Common | HE963725 | HE966863 |
| *Veronica serpyllifolia* L. | GenBank | Common | HQ590325* | FJ848101* |
| *Viburnum lantana* L. | GenBank | Common | JQ805391* | JQ805300* |
| *Viburnum opulus* L. | GenBank | Common | JN893691* | GU562393* |
| *Viburnum tinus* L. *tinus* | MIB:ZPL:03693 | Common | HE963726 | HE966864 |
| *Vicia cracca* L. | MIB:ZPL:03424 | Common | HG417059 | HG800589 |
| *Vicia dumetorum* L. | GenBank | Common | JX505502* | JX505997* |
| *Vicia faba* L. | Genbank | Common | JN661200* | JX505998* |
| *Vicia hirsuta* (L.) Gray | Genbank | Common | JN893462* | JX506011* |
| *Vicia sativa* L. | Genbank | Common | JN661204* | JX506030* |
| *Vicia sepium* L. | GenBank | Common | JN661205* | JX506033* |
| *Vicia sylvatica* L. | GenBank | Common | JN891170* | JX506037* |
| *Vicia tetrasperma* (L.) Schreb. | Genbank | Common | JN661207* | JX506042* |
| *Vicia villosa* Roth | GenBank | Common | HM850464* | JX506046* |
| *Vinca minor* L. | MIB:ZPL:03133 | Common | HE963729 | HE966867 |
| *Vincetoxicum hirundinaria* Medik. | MIB:ZPL:03048 | Common | HE963731 | HE966869 |
| *Viola canina* L. | MIB:ZPL:03388 | Common | HE963732 | HE966870 |
| *Viola hirta* L. | MIB:ZPL:01712 | Common | FR865127 | FR865081 |
| *Viola mirabilis* L. | GenBank | Common | GQ262539* | GQ262602* |
| *Viola reichenbachiana* Jord. ex Boreau | MIB:ZPL:02020 | Common | FR865134 | FR865088 |
| *Viola tricolor* L. | MIB:ZPL:03463 | Common | HG417060 | HG800590 |
| *Vitis labrusca* L. | MIB:ZPL:03993 | Alien | HE963734 | HE966871 |
| *Vitis vinifera* L. | MIB:ZPL:03391 | Common | HE963735 | HE966872 |
| *Vulpia myuros* (L.) C.C. Gmel. | GenBank | Common | FN870411* | HQ600143* |
| *Wisteria floribunda* (Willd.) DC.. | Genbank | Alien | AB729100* | EU424098* |
| *Xanthium strumarium* L. *strumarium* | GenBank | Common | DQ006070* | DQ006156* |
| *Xerolekia speciosissima* (L.) Anderb. | MIB:ZPL:04276 | Rare | HG417061 | HG800591 |
| *Zea mays* L. | GenBank | Common | Z11973* | GU575286* |
